# Unlocking Gen Z engagement in phygital retailing: Evidence from a multi-mediated customer experience model

**DOI:** 10.1371/journal.pone.0353490

**Published:** 2026-08-06

**Authors:** Mirlashine Selvendran, Sanidh Salman, Dulan Lakvidu, Udeera Dahanayaka, Krishantha Wisenthige, Nirmani Dayapathirana

**Affiliations:** Sri Lanka Institute of Information Technology (SLIIT), Malabe, Sri Lanka; National Institute of Technology, India (Institute of National Importance), INDIA

## Abstract

This study investigates how phygital experience influences Gen Z customer loyalty in Sri Lanka’s consumer electronics retail sector, addressing the limited evidence from emerging markets on hybrid retail approaches. The research emphasizes the growing relevance of integrating physical and digital touchpoints to create immersive consumer journeys and explores how such experiences foster loyalty among younger consumers. A quantitative research design was adopted using a structured 5-point Likert scale survey questionnaire was completed by 480 Gen Z customers of consumer electronics retailers in Sri Lanka. Data were analyzed through Partial Least Squares Structural Equation Modeling (PLS-SEM) to examine direct and indirect relationships between phygital experiences and loyalty. The study also evaluated a serial mediation mechanism to explain how immersive, technology-enabled environments and seamless physical–digital integration contributes to loyalty formation. Results demonstrate that phygital experiences significantly and positively influence customer loyalty. Gen Z consumers place high value on interactive, technology-driven retail environments that enhance satisfaction and strengthen their commitment to brands. The findings highlight the importance of co-creation, engagement, and perceived value in shaping loyalty, while underscoring the role of digital literacy in navigating hybrid retail contexts. The study provides actionable insights for retailers seeking to design effective phygital strategies that resonate with Gen Z consumers. It offers guidance for innovation and market expansion in emerging economies. However, the reliance on self-reported, cross-sectional data from a single sector and demographic limits generalizability. Future research should extend across industries, age cohorts, and cultural contexts, incorporating variables such as trust, emotions, and digital fatigue. This research contributes to the growing body of knowledge on phygital experiences by contextualizing the phenomenon in an emerging market setting. It advances theoretical understanding by empirically validating a mechanism linking phygital experience to loyalty and provides practical recommendations for retailers aiming to enhance customer journeys through hybrid strategies.

## Introduction

In the era where digital transformations rapidly shape human experiences, the boundaries between the physical and digital worlds are significantly dissolving. This convergence has given rise to the concept called “Phygital,” which was first founded by an Australian marketing agency called Momentum in 2013 [1]. The term phygital is widely defined as a hybrid concept that combines physical with digital elements and strategies to create interactive and personalized customer experiences [2,3]. For example, phygital tools that are involved in our day-to-day activities are basically contactless payment systems, interactive touch screens, and augmented reality, which make things more convenient [4–6]. This concept seamlessly facilitates day-to-day life, by making major changes in consumers’ behaviors and offering more dynamic connections between the physical and digital environments [7,8]. Moreover, while omnichannel and multichannel strategies are primarily focused on integration and management of distribution channels, the phygital customer experience (PH-CX) framework moves beyond this channel logic [4,9]. With this context, physical and digital elements are intentionally combined to create a seamless continuum of value across online and offline settings. This definition follows Batat’s conceptual framework, which positions phygital as a consumer-centered ecosystem rather than a simple channel strategy. Phygital is more than the mere connection of points of sale; it represents a holistic ecosystem that makes the customer journey seamless across physical and digital domains. Contrastingly, omnichannel emphasizes transactional continuity, phygital prioritizes a sustained continuous stream of values such as financial, symbolic, social, and affective which are delivered via hybrid experiences that fuse sensory design, technology, and experiential features [10,11]. Aligning with this, phygital should be operationalized as an experience construct, and not as a channel-management label. Even though this concept has rapidly evolved globally, as a whole, there is very little practicality among retailers and consumers. Researchers [12–14] have highlighted that the phygital concept is gaining traction, while widespread consumer understanding and adoption are still in early stages. By aligning with this fact, a developing country will face a situation on how to effectively accept and transform its businesses into a phygital experience-based store. However, the concern is that this concept has already been integrated with the consumer electronics retail sector [15], but such integrations by the retailer have happened without a proper understanding of the phygital concept and also without the intentional involvement of customers. The absence of practicability creates a problem in investigating the phygital concept in the Sri Lankan context.

Notably, phygital is an evolving ecosystem that can also be applicable for various sectors, such as the retail sector, education, fashion, banking, and insurance sectors [16]. Even though past literature has focused on sporting goods, banking, and other related sectors [17–19], they haven’t given much attention to the consumer electronics retail sector. Therefore, the main target audience of the study is customers using consumer electronics products such as smartphones, televisions, etc., which typically involve a combination of online information search and in-store evaluation prior to purchase. This blend of experience perfectly aligns with the concept of phygital, which integrates physical and digital touchpoints to enhance customer experience [20,21]. According to [22], the country (Sri Lanka) demonstrates a high level of digital readiness, amounting to over 12 million internet users, 29 million cellular mobile subscriptions and 21 million mobile broadband subscriptions. Hence, the widespread mobile connectivity suggests that consumers are routinely exposed to both online and offline touchpoints, enabling them to seamlessly interact with the mixed realms. Furthermore, [23] has stated that, consumer electronics have become an essential component that has a huge ability to shape our contemporary living and the way people interact with the world. Lastly, [24] has stated that the global consumer electronics market has a significant and sustainable growth and demand for the present, as well as in the future. Hence, this represents a problem in examining Phygital Experience (PE) behaviors in the consumer electronics retail sector. In addition to that, Gen Z are those who are purchasing and using consumer electronics products, also having the ability and the familiarity of using digital tools and technologies [25]. Existing literature [26,27] shows about targeting a consumer segment that engages heavily with technology and the internet can result in more precise insights and improved marketing approaches. Additionally, studies by [28,29] highlight that AR enhances the shopping experience of Gen Z by allowing them to visualize products in real-time and interact with virtual components. Retailers, who integrate AR technology into their business operations, such as virtual try-ons or interactive product demos can offer seamless experiences that fascinate Gen Z consumers to sustain their loyalty to the retailers. Aligning with this, the consumer electronics category is especially suitable because it includes products such as mobile devices, computers, and televisions, which commonly require online search, comparison, and in-store inspection before making the purchase. Therefore, it naturally reflects the phygital consumption patterns. Furthermore, due to the high Digital Literacy (DL) of Gen Z, they have a deep bond with the digital world, making them the majority of the age group using digital platforms for their buying decisions. Moreover, this represents a problem in examining the behaviors of Gen Z on PE.

Another important aspect is that when it comes to the empirical level, most of the prior studies have investigated the phygital concept by each variable relationship, for ex:- PE effects on Customer Engagement (CE), innovation, and consumer behaviors [30]. Even though past studies have done phygital-related studies, there is lack of studies that have investigated the impact of PE on major variables like DL, Customer Innovation (CI), Customer Co-Creation (COC), Perceived Value (PV), Customer Satisfaction (CS), and Customer Loyalty (CL) in one framework. Furthermore, the study focuses on combining eight major variables in a single study.

Additionally, there are fewer studies which have investigated the mediating impact of digital literacy on PE and CE. Although the existing study [30] indicated about the impact of PE on CE. There is a lack of studies that have given attention to the mediating role of DL on PE and CE. Lastly, the study focuses on addressing this mediation relationship, which prior research has identified as rare and in need of further investigation.

Afterwards, with the slant of these problems, the study carries the objective to investigate the impact of PE on CL through variables like DL, CE, CI, COC, PV, CS, and CL. The addressed gaps contribute to the growing field of PE literatures by providing existing PE models with new novel variables like DL, COC, PV, CS, and CL; focusing on the consumer electronics retail sector and evaluating Gen Z’s behaviors and engagement towards PE; assessing the mediation of DL on PE and CE to understand the consumer’s ability to navigate digital technologies.

The next section presents the literature review, followed by the methodology section, data analysis section, discussion, implications, and conclusion sections. The article ends with policy implications, limitations, and directions for future research, followed by the conclusion.

## Literature review

**Fig 1:**
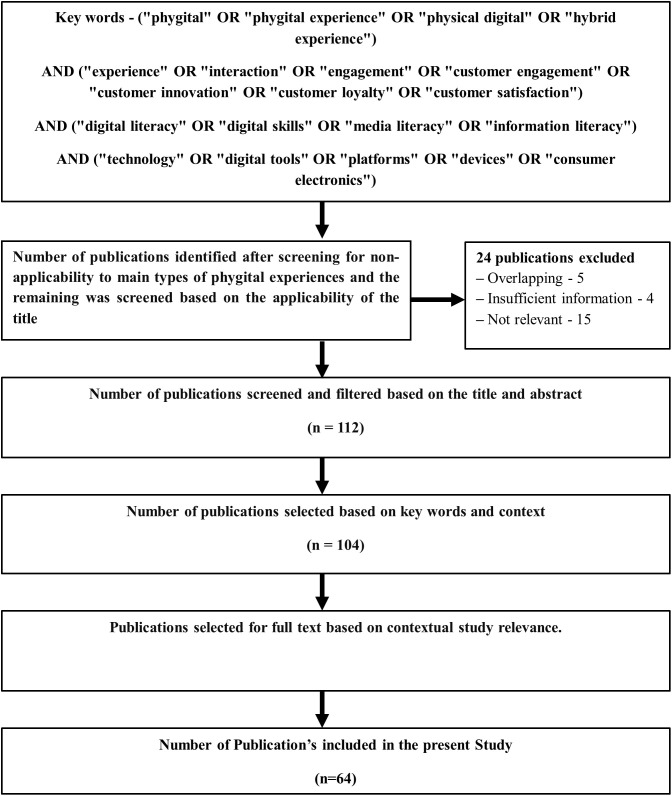
Prisma search flow diagram.

For this investigation, studies related to PE published between 2018 and 2025 were referred to. The search strategy led to the identification of a full-text publication available online. The process of selection involved searching both titles and abstracts to choose articles suitable for the study. While excluding irrelevant articles, 64 articles were screened for the current study. The Prisma flow diagram for the literature review is shown in Fig 1- Prisma Search Flow Diagram.

### Theoretical conceptualization

The Technology Acceptance model (TAM) proposes that adoption of technology is driven by Perceived Usefulness (PU) and Perceived Ease of Use (PEOU) [31,32]. It reflects variants such as TAM2 by [33] introducing additional predictors which include subjective norms and job relevance, where TAM3 [34] further highlights additional elements such as computer self-efficacy, anxiety, enjoyment, and usability. This widens TAM3 to use beyond technological environments and to diverse user communities such as hybrid learning [35]. Complementing TAM3, Service Dominant Logic (SDL) introduced by [36] describes values as COC, which does not exist in a purely physical form. With this, SDL also aligns with PE, where customers actively co-create across digital and physical touchpoints [37], shaping personalized experiences which drive satisfaction and loyalty [38]. Moreover, TAM3 demonstrates a close view to understand individual opinions such as PU, PEOU and DL which drive towards CE along with PE. In contrast, SDL enhances this perspective through embedding the adoption drivers within a holistic service ecosystem where customers serve as operant resources, value co production via CI and engagement. TAM3 constructs are viewed not solely as drivers of technology acceptance but also a mechanism of co production of value, reflecting SDL’s emphasis on resource integration. Together, TAM3 and SDL provide a great deal in regard to customer adoption and experience in phygital contexts.

### Phygital experience and customer engagement

Today’s modern consumers are looking for more immersive and meaningful interactions that go beyond simply buying products. With the growing demand for engaging experiences [18,39], companies are increasingly adopting hybrid product marketing strategies that combine digital and physical elements to attract and captivate consumers more effectively. Recent studies [37,40–43] have defined PE as hybridizing physical and digital aspects to create a unique customer experience at the same time and in the same atmosphere. PE obliges businesses in blending in-person and virtual encounters to secure client interactions while seeking distinctive involvement [17].

Notably, [41] emphasizes that companies are improving customer journeys by delivering personalized and dynamic experiences powered by technologies like Augmented Reality (AR), Virtual Reality (VR), and the Internet of Things (IOT), bringing innovation, and speediness to every interaction while generating a more holistic experience. Studies by [44,45] show that PE and CE have a positive relationship. PE would emphasize CE by creating a flawless, emotional experience that nourishes intense brand interaction [7]. As mentioned, PE fosters CE by blending digital and physical touchpoints, where TAM3 supports this link through perceived enjoyment and usability drive engagement.

Furthermore, TAM3 highlights that PU and PEOU are core to determine CE through technology. For instance, when users experience an innovative service setting, their level of involvement is identified by the extent to which they understand interaction as relevant, seamless, and compatible with their expectations [46]. TAM3 delivers justification at the operational level, where CE arises as a response behavior towards positive intellectual reviews of the system. By implementing TAM3, the relationship is reinforced through the reasoning that positive user perceptions of technology can directly translate to greater extent of CE. Based on the above discussion, the following hypothesis is proposed.

H_1_: Phygital experience has a significant impact on customer engagement.

### Digital literacy

While basic reading and writing remain valuable, DL has become essential for all. According to [47], DL is being defined by a wider range that goes just beyond technological competence, and is being substituted by media literacy. It also emphasizes that DL is the everyday skills that are needed to benefit from digital technology, to navigate information, and to be social. Furthermore, DL is a strategic ability that enables customers to respond and utilize digital tools effectively. The mediating role guarantees that PE transforms into deeper level of engagement, where customers who are tech–savvy can better recognize opportunities in dynamic environments. Furthermore, it also confirms that DL functions as a highly influential mediating construct in between digital capabilities and business outcomes, which highlights its importance in contributing to engagement [48]. Another study by [49] has said that DL is the ability to effectively use online content, tools, and resources. A study by [47] has proved that consumers’ DL clearly determines their buying patterns and their engagement, and the relationship confirms that DL plays a crucial role in enhancing CE.

Moreover, [50], have identified that customers with high digital technology knowledge will increase the possibility of them buying from the brand repeatedly, and it is because customers with a high DL tend to form a stronger self-efficacy and consider digital interactions as less complex. In TAM3, self-efficacy reduces perceived difficulty and increases the PEOU, thereby elevates the overall effectiveness of PE, where customers tend to purchase repeatedly from the same brand [51]. Hence, this improved self‑efficacy paves the way for more favorable cognitive evaluations of usefulness and ease of use, which directly promotes engagement behaviors [52]. This way, TAM3 provides theoretical justification for H_2_ by exhibiting that DL acts as a cognitive enabler, mediating the effect of system experience on CE. Existing studies have largely ignored the Phygital involvement of customers who are at different DL levels though very limited research has been done on how digital competency affects behavior. Accordingly, from the given literatures, the hypothesis is derived.

H_2_: Digital literacy mediates the relationship between phygital experience and customer engagement.

### Customer engagement and customer innovation

CE refers to the psychological involvement and thoughtful dedication a customer shows toward a business or brand [53]. Active CE exceeds beyond transaction-based involvement to sharing ideas, feedback, self-initiated actions in a manner that benefit both the customers and organizations. In SDL, such integration of resources can enable the organization to co-create value and drive towards CI, where active participation does not only benefit organizations, but enriches the overall output within the community [54] CE is best described as an ongoing exchange and interaction between businesses and their customers, which fosters deep psychological, emotional, and behavioral connections among brands, organizations, and their consumers [55]. Moreover, [56] have mentioned that CE is a factor that emphasizes customers’ preference towards products and in return it enables customers to accept the changes the organization has made and facilitates innovation by involving the feedback process.

In another study by [43], the scholars have put forward that CE is being used as a boost approach towards competitive advantage, which is an antecedent to innovation and COC.

Furthermore, [57] has explored that CI is the present setting, in an anticipatory behavior by customers, which impacts both product and service. Research also suggests that most customers are becoming active co-producers and are no longer a passive audience. Not only that, but sometimes online CE can also be stressful, as proven by [58] that it can create information overload, resource depletion, and customer stress rather than enhancing it. SDL frames that CE as a precursor to innovation and also it emphasizes that active participation in activities enables new value and improvement to service.

CE also represents a customer’s ability and willingness to invest time, effort and knowledge in interactions and SDL emphasizes that such resource combination is the foundation of innovation in service ecosystems [59]. Thus, SDL shows that CE is a vital antecedent of CI, as it enables the active participation and knowledge sharing necessary for COC [60]. With that, this study focuses on both physical and digital experiences. Past studies [61] have stated that phygital strategies make customers more engaged by capturing their attention and involvement through leveraging interactive technologies. The foregoing discussion culminates in the following hypothesis,

H_3_: Customer engagement has a significant impact on customer innovation.

### Customer innovation and co-creation

CI has emerged as a transformative concept in marketing and innovation literature, reflecting a shift from firm-centric innovation models to more participatory, customer-driven approaches [62]. According to [63] CI can be interpreted as an active involvement of customers in the idea creation, development, and refinement of products and services. The customer COC, is a collaborative and interactive process where multiple actors, like customers, firms, and users, integrate resources or knowledge to jointly create value, come up with new products, outcomes, or meanings by not just consuming them [57,64]. According to [65,66], customers’ innovativeness is an inherent trait or a perception that significantly boosts their participation in value COC in the phygital context. CE and CI are key drivers for patronage intention on phygital shopping platforms. CI indicates engaged participation where customers share insights, form ideas, and collaborate with organizations. This user driven behavior not just encourages new solutions, but it also enhances purchase intentions, where customers develop stronger connection to the end results which they helped shape [30]. Within SDL, knowledge and creativity turn into a service ecosystem, in which their participation translates CI to COC, by enhancing organizational values and customer loyalty [67]. Moreover, when customers innovate, they extend the scope of resource integration by introducing novel approaches that reshape interactions and enhance collective outcomes [68]. Where, SDL identifies that CI is not an isolated activity but a critical antecedent of COC within service ecosystems. Notably, [69] have stated that modern consumers actively shape their own experiences through digital interactions by giving importance to collaborative value creation between businesses and customers. From the above reasoning, the hypothesis is stated as follows,

H_4_: Customer innovation has a significant impact on co-creation.

### Co-creation and perceived value

Co-creation presents an active agency of customers who shape products, adding greater depth to insights. [70] show that when customers are involved in co-creating programs, those programs become more authentic, as a result, people value the brand more, which can raise the brand equity. [71,72] exploit the DART framework (Dialogue, Access, Risk evaluation, Transparency) because each component supports the brand experience, which enhances the satisfaction level in COC. In return, it raises the PV, where customers see the outcomes which they helped shape. In the meantime, SDL directly tries COC to value perception, and then SDL declares that value is not deep-seated in products or services, but is co-created through interactions among active participants in an integrated service network [36]. Moreover, SDL highlights that PV is a relational construct emerging from the COC process rather than being delivered one-sided by firms [73]. Further, [74,75] in this digital world, people don’t just shop passively, but they also collaborate, share ideas, and give feedback to shape the product at the end. This shift shows us that the customer plays a central role in value co-creation. Based on the above explanation, the hypothesis is built below,

H_5_: Co-creation has a significant impact on perceived value.

### Customer engagement and perceived value

According to [76], PV refers to a customer’s favorable assessment where the advantages of a service, both practical and experiential, exceed the associated cost or effort. Phygital elements such as AR, VR, seamless payments, and interactive displays offer relevance, reliability, and personalization, as a result, it heightens customer PV by merging both enveloping experience with convenience [77,78]. A study by [30,78] highlights that integrating innovation and personalization into phygital elements drives CE, which in turn significantly increases customer retention and patronizes the retailer. This has also been proved in digital services, where research in mobile app environments has been published by [79] showing us that PV includes quality, hedonic enjoyment, social value, and fair price, has a positive influence on engagement. [80] identifies that engagement in most cases is mediated by the impact that is made by the PV dimensions, and this illustrates the behavioral and psychological bridge.

Lastly, [81] have proved that the presence of interactive tech features attracts customers and enhances their perceived in-store values, with this the retailers can leverage these tools to captivate customers and build up engagement. Lastly, TAM3 emphasizes that PU and PEOU are crucial to shape user evaluations of technology; it also explains that CE reflects the behavioral manifestation of these positive cognitive evaluations, as individuals who perceive the respective system as useful and intuitive, are more likely to invest effort and attention in their engagement [82]. Such engagement enhances the value perception because customers experience outcomes perfectly align with their expectations and deliver efficiency and effectiveness. This way, TAM3 explains that PV emerges as a direct consequence of engagement behaviors rooted in favorable cognitive assessments of usefulness and ease of use. Based on the above literatures, the hypothesis is proposed below,

H_6_: Customer engagement has a significant impact on perceived value.

### Perceived value and customer satisfaction

[83] have identified in a systematic literature review that consumers’ PV exceeds their initial expectations when the product or services are more satisfying. [84,85] investigates entertainment platforms and finds that utilitarian, hedonic value, and social value factors significantly enhance satisfaction and show the strongest mediation effect. PV is also enhanced by service quality constructs such as responsiveness, confidentiality, and reliability, and not solely by co-creation. The evidence is supported from an e-banking study showcasing efficiency, reliability, responsiveness and confidentiality; it all indicates positive and significant effect on e-banking services, which in turn inferred a strong positive impact on CS [86]. Furthermore, in online shopping [87], the study shows that when customers experience a wide product range, accurate information, and convenient delivery, they perceive greater value in their interactions, therefore increases their satisfaction.

When most customers feel satisfied about what they have purchased and how well they have been treated, they are more likely to be retained and repurchase the same product [88]. Good customer service, real-time quick support, and especially a user-friendly system reduces the barriers to shopping, thus it builds brand trust while SDL emphasizes value experiential as a driver of satisfaction and conceptualizes that value is co-created through interactions among active participants rather than delivered unilaterally by firms. CS thus emerges as the affective consequence of PV, reinforcing SDL’s proposition that value creation is inherently tied to customer participation and COC processes [89]. Reflected on the above given information, the following hypothesis is proposed,

H_7_: Perceived value has a significant impact on customer satisfaction.

### Customer satisfaction and customer loyalty

As mentioned above, if customers feel more satisfied with the product or service that exceeds their expectations, then it would lead those customers to patronage intentions and to an emotional attachment too [90]. Satisfaction fully mediates the relationship between perceived efficiency and loyalty, which is identified in an Artificial Intelligence (AI) powered customer service context, thus, this shows satisfaction’s gains are transforming to loyalty [91,92]. SDL also explains that satisfaction is an antecedent of loyalty for exchange in service. It also serves as an empirical foundation for long-term relationships, where satisfied customers identify that their anticipated outcomes have been met through operant resource integration, which consolidates their intention to continue the relationship. Therefore, SDL shows that CL is a logical progression of CS.

Furthermore, a study by [93] illustrates that satisfaction is one of the 4 core drivers in influencing loyalty and consistent impact across geographies and industries. Finally [94] also showcase that in the omnichannel retail sector, the seamless flow across both physical and digital channels is because of satisfaction and the experience with trust and consistency by the customer, Moreover, in phygital settings, the direct path is crucial; as explained previously, if customers perceive that omnichannel experiences sustain performance aligned with expectations, they tend to maintain a lasting relationship. Through empirical findings in digital banking proves that CS, along with generative AI chatbots, explicitly boosts loyalty outcomes by consolidating confidence and sustained adoption [95], which strongly drives towards loyalty. From the above synthesis, the following hypothesis emerges,

H_8_: Customer satisfaction has a significant impact on customer loyalty.

The serial mediation role of DL, CE, CI, COC, PV, and CS in the impact PE on CL is examined. DL allows customers to engage with phygital channels effectively, which in return drives towards CE. CE fuels innovation and customer COC activities, where both are key in molding customer – PV and CS. The given multiple serial mediation approach incorporates cognitive readiness, emotional engagement, and customer participatory activities, offering a strong explanation of how phygital experiences translate into loyalty. By representing these mediators jointly, the framework ensures there is no critical dimension is overlooked [57,96]. Ultimately, the proposed mechanism identifies the multi – faceted understanding on how CL process, and how the contribution come along together to build the lasting loyalty. TAM3 identifies that DL strengthens PU and PEOU, by facilitating customers to engage assertively with phygital systems. This cognitive structure triggers engagement that transitions to SDL, where engaged customers facilitate CI and COC. When customers gain greater value, it strengthens CS as a positive result [73]. Thus, TAM3 supports the initial integration point of DL and CE, while SDL explains the connection chain leading to CL. Based on the prior literature, the following hypothesis formulated,

H_9_: Digital literacy, customer engagement, customer innovation, co-creation, perceived value, and customer satisfaction significantly serially mediate the impact of phygital experience on customer loyalty.

The serial mediation role of DL, CE, PV, and CS in the impact of PE on CL is examined. DL helps customers to feel confident and capable enough to engage with phygital channels, which enables them to grab digital technologies entirely. Therefore, it has a positive effect on CE where customers who are digitally literate are presumably engaged in both offline and online channels. Furthermore, engagement has a significant impact on CL, which is a result of PV, where this plays a critical role in determining CL. Therefore by locating DL in the mediation chain, it emphasizes that loyalty is not only an emotional connection product but also of technological fluency and readiness cognitive [79,96,97]. These joint mechanisms identify how loyalty in phygital context is being strengthened by customers both digitally and emotionally. The relationship justifies TAM3 at the entry point of DL, where DL fortifies self-efficacy and weakens perceived complexity by enabling customers to confidently interact with PE. Additionally, SDL labels proactive involvement as resource incorporation which enhances PV driving it to CS in response to co-created outcomes and sustaining CL [2]. Based on the above scenarios the hypothesis is given below,

H_10_: Digital literacy, customer engagement, perceived value, and customer satisfaction significantly serially mediate the impact of phygital experience on customer loyalty.

The serial mediation role of CE, CI, COC, PV, and CS in the impact of PE on CL is examined. PE motive CE by personalized and interactive touchpoint experiences. Engaged customers are more likely to move on to more behaviors which are innovative, idea generation and experimental, which extends their participation beyond consumption. Innovation always translates into COC, and customers directly participate in service and product development. COC also increases the perception of value by ownership and relevance, which results in satisfaction and loyalty. Through serial mediation, all the behaviors describe the complete process of customers as active participants [97–99]. This process reiterates on how engagement transforms into active participation and then back to loyalty through COC and satisfaction. SDL supports this serial mediation, [100] explains that CE functions as a catalyst for integration of resources where it encourages CI within service delivery. CI gradually transforms into COC, in which customers work together with firms to tailor experiences to meet user needs [57]. These co-produced outcomes tend to increase PV, and higher the PV it increases CS where satisfied customers are more likely to be loyal [97]. Therefore, the hypothesis below is developed,

H_11_: Customer engagement, customer innovation, co-creation, perceived value, and customer satisfaction significantly serially mediate the impact of phygital experience on customer loyalty.

The serial mediation role of CE, PV, and CS in the impact of PE on CL is examined. PE integrates technology with experiences which are tangible to provide an engaging environment which boosts customer interactions. Although engagement not only fosters immediate interaction, it also affects value perception, on how the consumers perceive this combined experience to be more personal, streamlined, and rewarding. PV is the most essential driver of satisfaction, and it significantly influences loyalty which has been endorsed by vast bodies of research. The above-mentioned variables suggest a sequential psychological journey where CE acts as the driver for satisfaction which ultimately arrives to achieving long term loyalty [101–103]. This pathway shows us how loyalty is not formed alone but it emerges through an ordered series of evaluative stages and experiential. SDL assists this serial mediation because CE elevates PV, since customers examine the end results more optimistically. Hence, they identify their role is shaping the outcomes, thus increases CS leading towards a long-term CL [104]. From the above discussed information, the hypothesis below is derived.

H_12_: Customer engagement, Perceived value, and Customer satisfaction significantly serially mediate the impact of phygital experience on customer loyalty.

**Fig 2:**
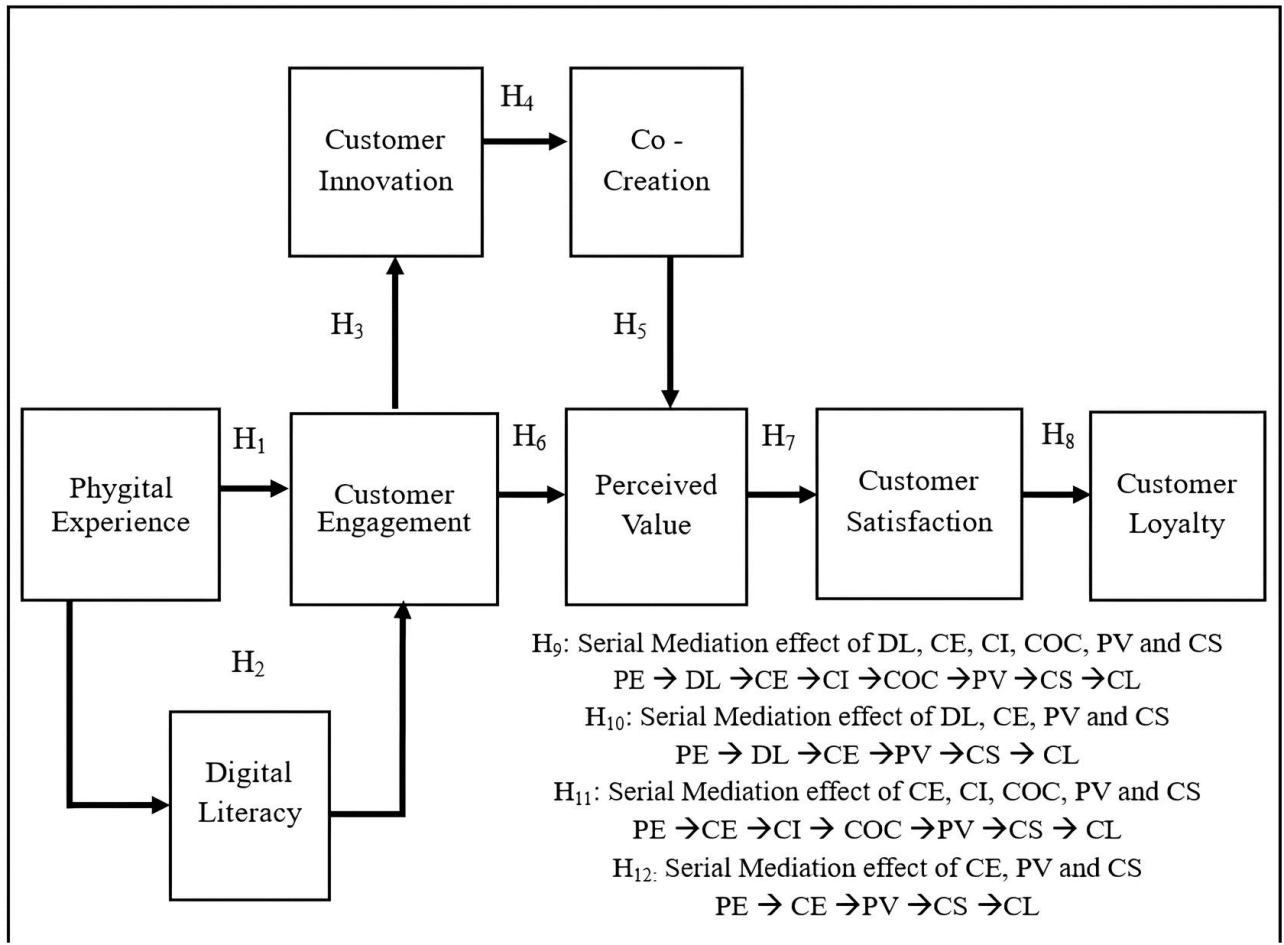
Conceptual framework.

Conceptualization is developed based on many literatures of conceptual foundations and formalized theories shown in Fig 2 – Conceptual Framework.

## Data and methodology

### Methods

The study primarily focused on investigating the factors influencing PE on consumer electronics retail stores using a quantitative empirical research design, grounded in a positivist philosophy and deductive reasoning method [105]. A structured survey was conducted online, measuring variables using a 5-point Likert scale ranging from “Strongly Disagree – 1” to “Strongly Agree – 5” based on previous research [13,106] to understand customer engagement behaviors (CEB) towards the phygital concept, focusing on observable phenomena and statistical analysis for generalizable conclusions.

### Population and sample

The target population of the study was Gen Z customers who purchased electronics products from consumer electronics retailer shops, highlighting the influence of digital technologies on shopping behavior and sales. Sri Lankan Gen Z purchasing behaviors are more blended with digital technologies [107]. Not only exposed to digital interactions but also to both digital and physical or Phygital interactions. The purposive sampling method, which was used by [18] helped to capture the respondents who experienced phygital aspects in their buying process, and the sample consisted of 480 respondents. This study specially targeted respondents who engaged with phygital experience related activities during their shopping journey. Through the study, authors aim to examine customer engagement and innovation behaviors within the specific content. The selected sampling approach enables the selection of respondents with relevant experience, thereby improving the validity of responses.

### Data collection

Data collection was aimed at collecting and analyzing numerical data to test hypotheses and determine relationships among the variables [108]. The survey questionnaire fulfilled requirements and qualifications to do an investigation, the online method was chosen for cost-effectiveness, ease of distribution, and suitability for urban areas [109]. For this, QR code scanning facilities was used to distribute the survey link, allowing respondents to easily access and complete the questionnaire using their mobile devices. Despite this, the use of QR code-based online data collection may have influenced the sample composition by identifying the respondents who are more digitally literate and more comfortable with mobile-based interactions. Simultaneously, the sample may have overrepresented digitally connected Gen Z consumers, while underrepresenting individuals with low levels of digital access or familiarity (i.e., digital divide).

### Measures

The primary goal of the study was to investigate the impact of PE and CL through CE, CI, COC, PV, and CS with a DL considering as a main mediative variable. Instead of CI and COC that played mediative roles between CE and PV to investigate the above-mentioned casual relationships from the correct target population, the questionnaire relied on the three (3) main layers. The first layer included the demographic information of the participants of the survey, the second layer had four (04) main questions for filtering known as purposive questions. “1. Have you used services/products that combine physical and digital experiences?”, “2. Have you ever given feedback or suggestions about a product or service you purchased?” “3. Have you purchased products or services from the brand/shop/retailer in the last 6 months?”, “4. Have you recommended a product or store to someone else based on your experience?”. The final layer consists of dimension validation questions with a 5-point Likert scale. These second and final layers were modified according to the contextual situation and improved practicality from the customer’s perspective. Before finalizing the survey questions, a pilot test with 70 responses was conducted to confirm the questions and their practical usability in customer perception. Then, inconsistencies were identified, and questions were re-modified to obtain a valid and reliable output. Table 1 used for building the questionnaire represents the indicators of the variables.

**Table 1:** Operationalization table.

Variable	Definition of the variables	Dimensions	Item	Source
PE	The blending of physical and digital elements enhances a customer’s shopping experience and their preference for such an integrated electronic retailer’s environment.	• Sensory Experience	PE1	[13,106,110,111]
		• Social Experience	PE2	
		• Emotional Experience	PE3	
		• Cognitive Experience	PE4	
		• Technological Experience	PE5	
DL	Confidence and ability to effectively utilize digital tools and platforms for shopping-related activities and information access.	• Information and communication technology	DL1	[112,113]
		• Media Literacy	DL2	
		• Information literacy	DL3	
CE	The extent to which customers interact with and feel connected to the shop, both online and offline, influences their behaviors.	• Cognitive Engagement	CE1,CE2	[106,110,114]
		• Affective Engagement	CE3,CE4	
		• Behavioral Engagement	CE5,CE6	
CI	Customer openness to adopting new shopping methods introduced by the electronic retailers, and the opportunities provided by the retailer for customer participation in idea generation or feedback.	• Innovation in services	CI1	[115,116]
		• Managerial innovation	CI2	
		• Innovation in process	CI3	
COC	Customers’ belief that their feedback and ideas are valued by the retailer and that they are involved in shaping the products or services they use.	• Who actually participated in the co-creation process?	COC1,COC2, COC3	[117–119]
PV	Customers’ assessment of the benefits received from products or services in relation to their cost, including their satisfaction with product quality, uniqueness, and after-sales services.	• Social Value	PV1	[114,120–122]
		• Emotional value	PV2	
		• Functional value	PV3,PV4	
		• Conditional value	PV5	
		• Epistemic value	PV6	
CS	Customer’s overall contentment with the product/service quality, the value received, and whether their expectations were met or exceeded, leading to a positive feeling about their choice.	• Satisfaction toward quality	CS1	[88,117,120,123]
		• Satisfaction toward value	CS2	
		• Conformity of expectation	CS3	
CL	A customer’s commitment to repurchase, recommend and stay with a brand, this is also driven when customer’s meeting exceeds their expectations.	• Word of Mouth	CL1	[123–125]
		• Repurchase Intention	CL2	
		• Customer Retention	CL3	

Source: Authors’ compilation.

### Ethical consideration

To ensure ethical content, this investigation aimed to protect participants’ rights and maintain record integrity. The team explained the study objectives, roles, questionnaire, and data management methods. Data were anonymized, protected from unauthorized access, and restricted to approved team members. Voluntary participation was encouraged, and data security and truthfulness were prioritized. Discipline and secure uploading of data were crucial for research success. Ethical approval was granted for the data collection by the Sri Lanka Institute of Information Technology (SLIIT) Business School Ethics Review Committee.

### Data analysis

The data was analyzed using quantitative methods to understand the relationship between variables, and data cleaning was ensured by removing all missing values, outliers, and inconsistencies. The Structural Equation Modeling (SEM) with Partial Least Squares (PLS) was used to analyses via SmartPLS 4.1.1.4 software, a suitable technique for explanatory models involving multiple constructs, and dimensions [126]. Apart from other analytical techniques, PLS-SEM model aligns with research objectives, fits data characteristics and suits model complexity. The analysis included a measurement model for reliability and validity, and a structural model for the strength and significance of relationships. The results validated conceptualization and provided empirical evidence on how PE influences CE, PV, CS, and CL. Following [127], serial mediation allows the examination of causal chains involving more than two mediators, where the effect of an independent variable is transmitted sequentially through multiple mechanisms and the empirical evidence remains limited on how phygital experience influences CL through multiple, ordered mechanisms involving DL, CE, CI, COC, PV, and CS.

## Results

### Demographics

The study reveals a diverse demographic, with a majority of respondents were male 56.67% while females accounted for 43.33%, indicating fairly balanced gender representation. Young adults dominate with 66.67% aged 22–25. Most respondents have a bachelor’s degree or undergraduate, with 86.88% having advanced degrees. The highest occupation is students, with 26.88% employed and 6.88% self-employed. Furthermore, 67.29% of respondents are having less than LKR 50,000 income. Exploring that monthly (29.79%) and rare (35%) purchases were most common, with 87.29% of respondents engaging in phygital experience during their buying process. Along with this, 419 out of 480 respondents were taken into the analysis due to the minimum requirement subject. This is because the study only considers valid data from respondents who have gained phygital experience during their shopping journey. Table 2 details the full demographic analysis of the respondents.

**Table 2:** Demographic information.

Measure	Item	Count	Column N %
Gender	Male	272	56.67%
	Female	208	43.33%
Age	18–21	130	27.08%
	22–25	320	66.67%
	26–28	30	6.25%
Education Level	Advanced level (A/L)	33	6.88%
	Bachelor’s Degree/ Undergraduate	417	86.88%
	Diploma/ NVQ	15	3.13%
	Postgraduate Degree (Master’s/ PhD)	7	1.46%
	Up to Ordinary level (O/L)	8	1.67%
Occupation	Employed	129	26.88%
	Self employed	33	6.88%
	Student	311	64.79%
	Unemployed	5	1.04%
	Other	2	0.42%
Income Status	LKR less than 50000	323	67.29%
	LKR 50000–99000	83	17.29%
	LKR 100000–149000	43	8.96%
	LKR 150000–199000	14	2.92%
	LKR 200000 and above	17	3.54%
Frequency purchasing products at the relevant consumer electronic shop	Once a week	40	8.33%
	Monthly	143	29.79%
	Annually	129	26.88%
	Rarely	168	35.00%
Have you tried Phygital experiences that combine physical and digital experiences while shopping?	Yes	419	87.29%
	No	61	12.71%

Source: Authors’ compilation.

### Measurement model assessment

**Fig 3:**
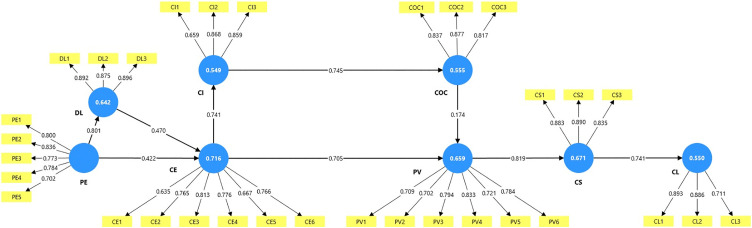
Measurement model analysis.

Through measurement model assessment the validity, and reliability of the latent variable and their outer loadings were evaluated. Table 3 represents 419 responses derived from the model assessment. In Fig 3 – Measurement Model Analysis, representing the measurement model analysis of conceptualization using SmartPLS4.

**Table 3:** Measurement model assessment.

Latent Variable	Item	Factor loading	Cronbach’s alpha	Composite reliability (rho_a)	Composite reliability (rho_c)	AVE
**CE**			0.833	0.843	0.878	0.547
	CE1	0.635				
	CE2	0.765				
	CE3	0.813				
	CE4	0.776				
	CE5	0.667				
	CE6	0.766				
**CI**			0.714	0.740	0.841	0.642
	CI1	0.659				
	CI2	0.868				
	CI3	0.859				
**CL**			0.782	0.826	0.872	0.696
	CL1	0.893				
	CL2	0.886				
	CL3	0.711				
**COC**			0.799	0.808	0.882	0.713
	COC1	0.837				
	COC2	0.877				
	COC3	0.817				
**CS**			0.839	0.843	0.903	0.756
	CS1	0.883				
	CS2	0.890				
	CS2	0.835				
**DL**			0.865	0.865	0.917	0.788
	DL1	0.892				
	DL2	0.875				
	DL3	0.896				
**PE**			0.838	0.842	0.886	0.608
	PE1	0.800				
	PE2	0.836				
	PE3	0.773				
	PE4	0.784				
	PE5	0.702				
**PV**			0.852	0.858	0.890	0.576
	PV1	0.709				
	PV2	0.702				
	PV3	0.794				
	PV4	0.833				
	PV5	0.721				
	PV6	0.784				

Source: Authors’ compilation using SmartPLS4.

According to the outer loadings of individual items, as recommended loadings greater than 0.70 are considered ideal, though values in the range between 0.635 and 0.70 can be acceptable because the construct demonstrated adequate average variance extracted (AVE) [128]. Most of the loadings were above 0.70, supporting indicator loadings. Except for a few items, such as CE1 (0.635), CE5 (0.667), and CI1 (0.659) exhibited relatively lower loadings. Aligning with the argument of [128,129], those items were retained due to their acceptable acceptance level and satisfactory convergent validity (AVE > 0.50). Furthermore, these items were theoretically important in capturing the entire domain of the constructs, especially for multidimensional variables like CE and CI. Removing these items would compromise the content validity of the measurement model and using Cronbach’s alpha (α), composite reliability rho_a, and rho_c, internal consistency reliability was assessed. All constructs achieved reliability values above the recommended 0.70 threshold, with most exceeding 0.80, indicating good to excellent reliability performance [130,131]. At least having AVE at 0.50 is confirmed convergent validity, implying that more than 50% of the variance in the indicators is explained by the latent variable. The results indicated that all constructs satisfied this criterion, with AVE values ranging from 0.547 (CE) to 0.788 [132].

### Fornell-Larcker-criterion

In Table 4, the discriminant validity evaluation consists of the Fornell-Larcker criterion, which compares the square root of AVE values with the inter-construct correlations.

**Table 4:** Fornell-Larcker-criterion - discriminant validity.

	CE	CI	CL	COC	CS	DL	PE	PV
**CE**	0.740							
**CI**	0.741	0.801						
**CL**	0.704	0.654	0.834					
**COC**	0.537	0.745	0.512	0.844				
**CS**	0.716	0.648	0.741	0.499	0.870			
**DL**	0.808	0.679	0.606	0.448	0.640	0.887		
**PE**	0.798	0.655	0.598	0.441	0.643	0.801	0.780	
**PV**	0.798	0.724	0.738	0.553	0.819	0.715	0.687	0.759

Source: Authors’ compilation using SmartPLS4.

[133], each construct’s √AVE should exceed its correlations with other latent constructs, and results confirmed that the above condition was satisfied across all the constructs. This approach has been widely recommended in PLS-SEM literature [126] provided a reliable assessment of discriminant validity when HTMT ratios show potential issues. The √AVE for CE (0.740) and CS (0.870) showed higher correlations with CI and CL, indicating more variance with their dimensions. The measurement model results confirmed constructs’ reliability and validity, providing a robust foundation for structural model assessment.

### Structural model assessment

In structural model assessment, the focus is mainly on evaluating the statistical values to test the hypothesized relationships among the constructs. Path coefficients (β), standard deviation (σ), t-values, p-values, and effect size (f^2^) values analyzed, and derived decisions using the bootstrapping method in SmartPLS to determine the significance and strength of relationships. The results are represented in Table 5, indicate that all hypothesized relationships H_1_, H_3_, H_4_, H_5_, H_6_, H_7_, and H_8_ are statistically significant and confirm all the hypotheses. These findings indicate that the proposed model effectively explains the relationships between PE, CE, CI, COC, PV, CS, and CL, by demonstrating strong predictive capability for understanding CEB in phygital retail environments.

**Table 5:** Structural model assessment.

Hypothesis	Path coefficient	Standard deviation	t-value	p-value	f^2^-values	Decision
**H**_**1**_ **: PE → CE**	0.422	0.058	7.253	0.000	0.225	Supported
**H**_**3**_ **: CE → CI**	0.741	0.036	20.309	0.000	1.216	Supported
**H**_**4**_ **: CI → COC**	0.745	0.029	25.380	0.000	1.246	Supported
**H**_**5**_ **: COC → PV**	0.174	0.035	5.024	0.000	0.063	Supported
**H**_**6**_ **: CE → PV**	0.705	0.034	20.929	0.000	1.034	Supported
**H**_**7**_ **: PV → CS**	0.819	0.025	32.886	0.000	2.044	Supported
**H**_**8**_ **: CS → CL**	0.741	0.046	15..992	0.000	1.221	Supported

Source: Authors’ compilation using SmarPL4.

### Mediation model assessment

The mediation analysis is represented in Table 6, mainly focused on the effect of DL on the relationship between PE and CE was examined using the bootstrapping approach in SmartPLS. The results show that H_2_ relationship supporting the mediation role played by DL which has significant impact on the relationship between PE and CE ((β = 0.376, t = 7.369, p < 0.001). Digital skills significantly enhance engagement in phygital retail environment in phygital retail environments, with PE positively influencing CE through DL, emphasizing the importance of DL in strengthening PE’s impact. Furthermore, four significant serial mediation pathways were revealed, confirming that PE on CL has a positive and statistically significant in the first indirect mediation path through DL, CE, PV, and CS (H_9_: β = 0.022, t = 3.249, p = 0.001), through the second mediation path CE, CI, COC, PV, and CS in between PE and CE has positive significant (H_10_: β = 0.161, t = 5.591, p < 0.001), the third mediation path through CE,CI, COC, PV, and CS positively influences indirectly PE on CL (H_11_: β = 0.025, t = 3.570, p < 0.001) final and the fourth serial mediation through CE, PV, and CL (H_12_: β = 0.180, t = 5.641, p < 0.001) has a positive significant link. These findings confirm that PE enhances CL by sequentially strengthening CE, PV, and CS, while CI and COC further reinforce the CL formation process.

**Table 6:** Mediation model assessment.

Hypothesis	Path coefficient	Standard deviation	t-value	p-value	Decision
**Single Mediation**					
**H**_**2**_ **: PE → DL → CE**	0.376	0.051	7.369	0.000	Supported
**Serial Mediation**					
**H**_**9**_ **: PE → DL → CE → CI → COC → PV → CS → CL**	0.022	0.007	3.249	0.001	Supported
**H**_**10**_ **: PE → DL → CE → PV → CS → CL**	0.161	0.029	5.591	0.000	Supported
**H**_**11**_ **: PE → CE → CI → COC → PV → CS → CL**	0.025	0.007	3.570	0.000	Supported
**H**_**12**_ **: PE → CE → PV → CS → CL**	0.180	0.032	5.641	0.000	Supported

Source: Authors’ compilation using SmartPLS4.

### Collinearity values

The study tested collinearity through the variance inflation factor (VIF) of all dimensions, ensuring no multicollinearity bias. As shown in Table 7, the VIF values range from 1.177 to 2.364, below the conservation threshold of 3.3 [134] and the general threshold of 5.0 [126] indicate that multicollinearity is not an issue in this study. All dimensions were retained for subsequent structural model analysis.

**Table 7:** Collinearity Values (VIF).

Dimension	VIF	Dimension	VIF
CE1	1.363	CS2	2.171
CE2	1.704	CS3	1.753
CE3	1.982	DL1	2.331
CE4	1.785	DL2	2.069
CE5	1.440	DL3	2.364
CE6	1.737	PE1	1.960
CI1	1.177	PE2	2.172
CI2	1.845	PE3	1.732
CI3	1.800	PE4	1.660
CL1	1.996	PE5	1.443
CL2	1.974	PV1	1.615
CL3	1.365	PV2	1.598
COC1	1.693	PV3	2.016
COC2	1.837	PV4	2.220
COC3	1.634	PV5	1.559
CS1	2.144	PV6	1.856

Source: Authors’ compilation using SmartPLS4.

### Coefficient of determination (R²)

The co-efficient of determination (R²) evaluates the explanatory power of the structural model, in Table 8 representable of R² values. Results reveal that PE and DL account for 71.6% of CE variance, with CI and COC showing strong predictive power. PV is 61.5% and CS and CL show strong explanatory power. DL as a mediator has an R² of 0.642, exploring its substantial role in linking PE to CE. The adjusted R² values are very similar to the R² values, indicating minimal overfitting and confirming the robustness of the model. The PLSpredict procedure use to assess predictive validity, which measures the out of sample predictive performance of the model. The findings emphasize that all endogenous constructs show a positive Q^2^ prediction value by confirming the existence of predictive relevance. CE (0.634) and DL (0.638) both indicate strong predictive power with Q^2^ predict values exceeding 0.60, while CI, CL, CS, and PV show moderate predictive values. However, COC with a small predictive value (0.191), even though is negligible, demonstrates predictive capability. The overall results conclude that the model has strong explanatory capabilities in predicting key outcomes in phygital retail contexts.

**Table 8:** R², adjusted R², and Q^2^ predictive values.

	R-square	R-square adjusted	Q^2^ Predictive
**CE**	0.716	0.715	0.634
**CI**	0.549	0.548	0.421
**CL**	0.550	0.549	0.307
**COC**	0.555	0.554	0.191
**CS**	0.671	0.671	0.396
**DL**	0.642	0.641	0.638
**PV**	0.615	0.657	0.465

Source: Authors’ compilation using SmartPLS4.

### Model fit assessment

The SRMR value of the saturated model (0.071) is below the recommended 0.08 threshold [135], explored an acceptable fit, while the estimated model’s SRMR result (0.091) is slightly higher but still tolerable in PLS-SEM [136]. The d_ULS and d_G values (2.653) & (0.907), respectively, were low, suggesting a good alignment between the empirical and model-implied correlations. NFI values (0.767 and 0.757) show moderate fit, however, below the ideal 0.90 benchmark. Concluding the model fit, the saturated model provided a better fit, and the indices confirm that the model establishes an acceptable fit for PLS-SEM research. Table 9 represents values for the model fit assessment.

**Table 9:** Model fit assessment.

	Saturated model	Estimated model
SRMR	0.071	0.091
d_ULS	2.653	4.338
d_G	0.907	0.978
Chi-square	2119.978	2218.987
NFI	0.767	0.757
		

Source: Authors’ compilation using SmartPLS4.

## Discussion

The study focused on finding the impact of PE on CL while exploring the mediating effects of DL on PE and CE. Thus, the study was also conducted based on established theoretical frameworks, which are the Technology Acceptance Model3 (TAM3) by [34], and the Service Domain Logic (SDL) by [36].

The analysis results showed that PE had a significant impact on CE. This result mirrors the findings presented by [18,21]. Furthermore, [7] argue that customers’ journey in the phygital context, which can also be defined as hybrid artefacts, blended contexts, circular actions, and intertwined emotions, creates an immersive engagement loop that deepens customers’ involvement. When physical and digital touchpoints are seamlessly integrated into the journey, it leads to a stronger relationship between customers and brands. In addition, [137] also found that integrating AR, VR, IoT, and AI into physical retail spaces can notably enhance engagement, which offers a personalized and real-time experience. Similarly, [30] emphasize that phygital transforms the way businesses engage with their customers by combining the capabilities of digital technology and physical environments. Integration enables interactive customer journeys and immersive experiences. On the other hand, an earlier study by [138] identified that the ambiance of a physical store and staff behavior alone can influence engagement. But those models were developed before the wide adoption of digital technologies. Moreover, the possible causes of PE significantly impacting CE are emphasized by [7], that CE in a phygital context is circular and not linear, and it is driven by dynamic interactions across touchpoints. [137], further identified that immersive technologies retain attention and an emotional connection, which most of the traditional models fail to capture.

Next, the analysis proved that DL had a significant impact on mediating between PE and CE. Similar findings were identified in studies [30]. These studies empirically illustrate that their SEM results confirm that customers who have higher DL are more capable of navigating through the phygital retail environments. These consumers are also better at understanding the technologies that are being integrated into the journey. The significant mediating role of DL can be identified when customers effectively utilize the complex omnichannel experiences. In contrast, a customer with higher DL can easily adapt to the emerging technologies and manage seamless transitions between digital and physical [13]. In addition to that, [139] identify that phygital experience is being enhanced when digital solutions are added to physical environments. Moreover, DL enables consumers to effectively navigate, and understand how phygital retail technologies work [102]. This aligns with the conceptualization of PE as the integration of physical and digital elements, requiring technological skills for optimal interaction [140]. The factors identified in these prior studies-such as technological proficiency and customer cognitive experience, are fully aligned with the DL construct in this study, substantiating its role as a mediator.

Next, the analysis revealed that CE has a significant impact on CI. This finding confirms the past studies, which also presented similar findings in different aspects [30,56,115]. Conversely, a study by [141] show that a higher level of CE in innovation reduced customer complaints. When customers are engaged in innovation (such as participating in product development or giving feedback), they feel more connected to the product or service. This engagement leads to greater satisfaction because customers’ opinions and needs are considered, making them less likely to be disappointed or dissatisfied with the final product. Further, it has also been argued by [142] that CE in business activities builds a strong sense of relationship between individual customers and the entity. Through these strong social relationships, it enables the customers to willingly share their knowledge with the company. Therefore, the higher the customer engages with the business, the higher the chance for the customer to participate in the innovation process. Lastly, a study by [143] conclude that active CE positively and significantly affects service innovation and retail innovation. Engaged customers not only provide ideas and feedback for new products and services but can even reshape retail business models.

Furthermore, the study proved that CI has a significant impact on COC. [144] also found a similar result, and COC significantly influences the adoption of digital services, validating CI as a CE mechanism. Similarly, [145] suggest that users who are engaging are most likely to be involved in co-creating, and especially this happens in online communities, which helps them to be motivated through internal and external factors. Furthermore, [146] believe that the participation of customers in value co-creation can positively affect the well-being of customers. Their SEM results reveal that customers’ input is not marginal, but it is essential to CI and COC. [117] highlight that access to information, transparency, and feedback from customers play a vital role in strengthening COC. The results vary by identifying phygital ecosystems, where innovation is distributed, and customer input is essential. Moreover, [144] explain that CI is incorporated into ongoing COC loops, which explains the significant impact.

Next, this study revealed that COC has a significant impact on PV. This finding is strongly aligned with the prior studies. As witnessed by [147] have argued that co-creation has a positive impact on customers’ PV. Moreover, when there is a higher level of consumer involvement in value creation, it can lead to a better PV on the customer’s side. Notably, the study [147] have also identified that they have emphasized through a DART model, which is used to identify four key major blocks of co-creation that allow customer, and firms to co-create together. Also, another study by [148] has stated that in the process of value co-creation, it has a positive impact on customer PV. In phygital settings, co-creation might manifest as customers interacting with digital tools in-store to shape their experience or outcomes. Finally, customers who can customize products or services via digital tools in a physical store perceive higher utility and can learn and adapt to interactive technology. Lastly, these further establish the crucial role that COC can play in PV.

Furthermore, the investigations of this study have identified that CE has a significant impact on PV. This lines up with the prior study [123], which has revealed that CE indicates a significant impact on PV. Furthermore, [100] have explored that CE is a root of PV, and these studies investigated that when CE is high, the interaction between brands and customers becomes more meaningful. As a result, they will be more likely to perceive higher value from the products and services that they are purchasing and feel more satisfied with the process. Also, according to [149], when consumers feel they are getting enough benefits compared to their cost, they will engage more with the business, which proves that PV also strongly influences CE. [100] have identified that the main dimensions of PV, which are functional value and social value, are the most influential, while the emotional value doesn’t play a much stronger role with CE.

Next, the analysis revealed that PV has a significant impact on CS. This finding confirms the past studies, which have also shown similar findings in different studies [84,85,150,151]. In a study by [150] it has been emphasized that when the PV of a customer exceeds, then it positively impacts CS. In this study, they have also mentioned that PV plays a fundamental role in the relationship between CS and service. According to [151], PV plays a significant role in shaping CS. CS occurs when the price they paid for the product or service exceeds their expectations. This through a better customer PV can impact CS, which in turn gives a positive relationship.

The analysis has given findings that CS has a significant impact on CL. This finding adheres to [152], have stated that when examining the effect of CS on CL, a positive effect is shown between them. Also, the study has mentioned that CS is an indicator of the entity’s past, current, and future performance, and there is ample evidence for its positive effect on CL. Satisfying the customer by giving them an exceptional service is a major contributor to CL. The study has also revealed that CS is the key to success and further denotes that a satisfied customer is always a repeat customer. Moreover, if a customer is delighted about what he gets and if that goes beyond what he expects, then he is more than satisfied with that experience, and in return, stays loyal to that product or service.

The analysis has revealed that, the study has concrete evidence for a serial mediation effect of PE on CL through CE, PV, and CS. This shows the impact of PE on CL, which occurs through a structure psychological and experiential pathway. Moreover, Consistent with customer experience theory, integrated physical and digital touchpoints enhance interactivity and immersion, thereby stimulating higher levels of CE [100,153]. In line with the expectancy disconfirmation theory, enhanced PV subsequently leads to greater CS, in which the customers evaluate the benefits of PE outweighing the associated costs [149,154]. Hence, CS then acts as a critical affective driver of CL, reinforcing repeat purchase intentions and positive word-of-mouth. Lastly, [102,155] highlights that the presence of this serial mediation highlights that CL is developed progressively rather than instantaneously, underscoring the importance of managing PE as holistic, multi-stage journeys rather than isolated technological touchpoints.

Next, the analysis has revealed strong empirical evidence for a significant serial mediation effect, indicating that the influence of PE on CL operates through DL, CE, PV, and CS in a sequential manner. Furthermore, the result suggests that PE alone is insufficient to generate CL unless customers possess the digital competencies required to effectively interact with technology-embedded physical environments. Consistent with prior research [156], phygital contexts demand a baseline level of DL, which enhances users’ confidence, reduces perceived complexity, and enables more meaningful interaction with digital interfaces. Moreover, digitally literate customers experience lower cognitive effort and technology-related anxiety, they are more likely to actively engage with phygital touchpoints, supporting existing CE frameworks [100,157]. Lastly, [2] identifies that increased engagement subsequently facilitates value co-creation, allowing customers to better recognize functional, emotional, and experiential benefits, in line with service-dominant logic.

Next, the findings provide a strong empirical support for a significant serial mediation effect, demonstrating that the impact of PE on CL unfolds through CE, CI, COC, PV, and CS in a sequential manner. Recent customer experience research [102,158,159] emphasizes that phygital environments enhance interactive richness and customer participation by seamlessly integrating digital and physical touchpoints, thereby fostering higher levels of engagement. Notably, engaged customers are more inclined to contribute ideas, feedback, and creative inputs, positioning them as active participants in customer innovation rather than passive recipients of value. This innovation-oriented participation naturally evolves into COC, as customers collaborate with firms in shaping offerings and experiences, reinforcing service-dominant logic which conceptualizes value as jointly created through interaction [82,160]. Lastly [161,162], finds CS subsequently strengthens CL by reinforcing repeat purchase intentions and positive word-of-mouth behaviors making this finding valid.

The findings provide strong empirical support for a significant serial mediation effect, indicating that the influence of PE on CL unfolds through DL, CE, CI, COC, PV, and CS in an orderly manner. Digitally competent customers are more likely to actively engage with firms, and such engagement has been shown to stimulate customer-driven innovation through feedback, idea contribution, and creative involvement [163]. Through COC, customers perceive higher functional and experiential value due to increased personalization, relevance, and perceived control [164]. The integration of the serial mediation hypothesis was proposed to identify the integrated mechanisms through which PE determines CL. Each hypothesis symbolizes a separate yet complementary pathway. The expanded sequence in H_9_ shows how DL promotes CE, which then encourages CI and COC, eventually leading to PV, CS and CL. H_10_ tests whether DL and CE are solely capable of driving value and satisfaction, therefore it clarifies the role of CI and COC, while H_11_ identifies customer influenced mechanisms of CE, CI, and COC, which show that CL can appear even without DL as an initial step. Finally, H_12_ portrays the simplest SDL pathway, where CE directly affects value perception and CS, leading to CL. Together, these serial mediation hypotheses enable the model to differentiate between holistic and simplified processes, which ensure that mediators are not chosen randomly but are experimentally evaluated for necessity. The results validate that shorter chains represent essential dynamics, but the extended path provides a comprehensive explanatory power, by justifying the serial mediation hypothesis in the study.

### Managerial and social implications

In the Sri Lankan context, the integration of PE holds significant managerial and social implications. For Managers and retailers, it is essential to focus on blending digital and physical touchpoints through AR/VR showrooms, mobile-based technologies, real-time inventory tracking, and seamless payment systems to reduce adoption barriers and create immersive journeys that enhance engagement and loyalty [165]. Furthermore, strengthening consumer digital literacy is equally crucial in maximizing these benefits. From a social point of view, PE reshapes consumption standards through embedding smart technologies into everyday spaces, encouraging participatory cultures, collaborative innovation, and stronger social bonds [21]. Eventually, this integration not only improves CS and brand endorsement but also enhances consumer well-being.

### Limitations and future research

However, this research constructs a complex and meaningful model, this research has a few limitations. First, due to considering Gen Z respondents, there is a lack of analysis done on how other age groups engage with PE. Second, this research is based on quantitative self-reported questionnaires, which may introduce hidden bias or restrict deeper insights into customer behavior. Third, the research collects data at a single point in time, preventing analysis of changes or trends in CEB over time. Fourth, the research was limited to the consumer electronics retail store to specify the findings. Five, conducted only in Sri Lanka, comparing other countries, and cultural contextual results may differ due to cultural change, social change, and the rate of technological advancements. Finally, as the purposive sample technique chosen in this study may generate sample bias and limit generalizability, probability sampling techniques can be used for future investigations in broader populations. Even though, it has a few limitations, it clears a path for future researchers to explore more insightful investigations on PE and CL. Including different demographics extending to other industries in the retail sector, which suits a phygital framework, can help investigate the long-term effects of phygital strategies on other variables. Exploring newer variables like customer emotions, trust, and digital fatigue. Limitations and future research may lead to new paths for knowledge expansion, and developing the concept of physicality, integrating more with human interactions.

## Conclusion

The study contributes to the body of knowledge on hybrid retail ecosystems in an emerging market setting by providing empirical evidence that PE can be a strong driver of CL among Gen Z consumers in the consumer electronics retail sector of Sri Lanka. The PLS-SEM analysis inferred all the 12 hypothesized relationships. It means that PE has a positive and significant impact on CE, which in turn, activates the sequential value-creation chain through CI, COC, PV and CS and finally, leads to CL. One of the theoretical contributions of this paper is the empirical validation of DL as a significant mediator between PE and CE. This observation confirms that PE in isolation cannot achieve engagement unless the customers have sufficient digital skills to navigate technology-based retail space. Moreover, four serial mediation pathways established confirm that loyalty in phygital contexts is formed through a cumulative, multi-stage psychological process, but not through any specific, direct mechanism. In essence, these results provide pragmatic advice to retailers doing business in the emerging economies. Managers are not only to invest in the process of integrating AR/VR, IoT, and seamless digital/physical touchpoints, but also in building the DL of customers by providing in-store training, creating user-friendly interface design, and implementing technology onboarding programmers. PV and long-term loyalty can be further enhanced by fostering COC opportunities. This study adds to the extant literature of phygital by introducing new mediating variables in one integrated framework beyond previous studies on single mediating variables. It is also contextual to phygital adoption in a developing country like Sri Lanka (where political and economic instability are prevalent) and can be transferred to other emerging markets. However, the scope of the study is limited by the cross-sectional design, use of self-reported data, limited to one industry and a single demographic, as well as geographically to Sri Lanka. Future studies warrant the use of longitudinal designs and be extended to cross-cutting industries, age groups and even diverse cultural environments. The inclusion of such variables as consumer trust, emotional responses, and digital fatigue would further enrich theoretical frameworks of phygital loyalty formation.

## References

[1] Prats J. Adopting a retail phygital experience via open innovation: the story of WOW. Barcelona: IESE Business School; 2022.

[2] Hollebeek LD, Sprott DE, Andreassen TW, Costley C, Klaus P, Kuppelwieser V, et al. Customer engagement in evolving technological environments: synopsis and guiding propositions. EJM. 2019;53(9):2018–23. doi: 10.1108/ejm-09-2019-970

[3] Tichelen B. The role and opportunities of phygital in the digital omni-channel strategy. Belgium: Louvain School of Management; 2019.

[4] Batat W. What does phygital really mean? A conceptual introduction to the phygital customer experience (PH-CX) framework. J Strateg Mark. 2022;32(8):1220–43. doi: 10.1080/0965254x.2022.2059775

[5] Moravcikova D, Kliestikova J. Brand building with using phygital marketing communication. JOEBM. 2017;5(3):148–53. doi: 10.18178/joebm.2017.5.3.503

[6] Nofal E, Reffat RM, Moere AV. Phygital heritage: an approach for heritage communication. Third Immersive Learning Research Network Conference (iLRN2017); Coimbra, Portugal; 2017. p. 220–9.

[7] Mele C, Di Bernardo I, Ranieri A, Russo Spena T. Phygital customer journey: a practice-based approach. QMR. 2024;27(3):388–412. doi: 10.1108/qmr-08-2023-0102

[8] Vidarshika WO, Ranasinghe A, Dayapathirana N. A concept paper on impact of consumption values on patronage intention in phygital retailing: a theory of consumption values approach. In: Jayawardenapura UoS, editor. 21st International Conference on Business Management (ICBM 2025). University of Sri Jayawardenapura: Faculty of Management; 2025. 86 p.

[9] Yin CC, Chiu HC, Hsieh YC, Kuo CY. How to retain customers in omnichannel retailing: Considering the roles of brand experience and purchase behavior. J Retail Consum Serv. 2022;69:103070. doi: 10.1016/j.jretconser.2022.103070

[10] Sharma N, Dutta N. Omnichannel retailing: exploring future research avenues in retail marketing and distribution management. Int J Retail Distrib Manag. 2023;51(7):894–919. doi: 10.1108/ijrdm-05-2022-0166

[11] Gerea C, Gonzalez-Lopez F, Herskovic V. Omnichannel customer experience and management: an integrative review and research agenda. Sustainability. 2021;13(5):2824. doi: 10.3390/su13052824

[12] Maciejewski G, Wróblewski Ł. A typology of consumers based on their phygital behaviors. Sustainability. 2025;17(14):6363. doi: 10.3390/su17146363

[13] Liu L, Lee SH. Do people really care if it’s phygital retail? Exploring the relationship between customer experiences and customer behaviors. AMJ. 2024;26(3):186–200. doi: 10.53728/2765-6500.1637

[14] Batat W. Phygital customer experience in the metaverse: a study of consumer sensory perception of sight, touch, sound, scent, and taste. J Retai Consum Serv. 2024;78:103786. doi: 10.1016/j.jretconser.2024.103786

[15] Daily FT. Samsung opens first ever experience store in Colombo; 2018 [cited 2025 July 23]. Available from: https://www.ft.lk/Front-Page/Samsung-opens-first-ever-experience-store-in-Colombo/44-659271

[16] Alexander B. Customer experience in fashion retailing: merging theory and practice. 1st ed. London: Routledge; 2024.

[17] Bonfanti A, Vigolo V, Vannucci V, Brunetti F. Creating memorable shopping experiences to meet phygital customers’ needs: evidence from sporting goods stores. IJRDM. 2023;51(13):81–100. doi: 10.1108/ijrdm-12-2021-0588

[18] Kumar J, Rana S, Rani G, Rani V. How phygital customers’ experience transforms the retail banking sector? Examining customer engagement and patronage intentions. CR. 2023;34(1):92–106. doi: 10.1108/cr-04-2023-0076

[19] Testa DS, Slaton K. The future is phygital: integrated content analysis of current retail strategies and future priorities across digital, physical, and phygital modalities. J Retail. 2026;102(2):566–81. doi: 10.1016/j.jretai.2025.04.006

[20] Johnson M, Barlow R. Defining the phygital marketing advantage. JTAER. 2021;16(6):2365–85. doi: 10.3390/jtaer16060130

[21] Mele C, Spena TR, Marzullo M, Di Bernardo I. The phygital transformation: a systematic review and a research agenda. Ital J Mark. 2023;2023(3):323–49. doi: 10.1007/s43039-023-00070-7

[22] Kemp S. Digital 2025: Sri Lanka. DataReportal – Global Digital Insights; 2025.

[23] Yashas KSDBD. A study on consumer behavior on consumer electronics and home appliances of panasonic products at panasonic brand store, Naga Appliances, Bengaluru. IJFMR. 2023;5(5):1–12. doi: 10.36948/ijfmr.2023.v05i05.6275

[24] Erdem KR. A new perspective as a combination of physical and digital: phygital marketing. NOBEL BİLİMSEL ESERLER; 2022.

[25] Ranjith ADDP, Jayasingha JNM, Weerasekara ARK, Dayapathirana NN, Nisansala SPSF, Wisenthige K. Social media credibility on social commerce purchase intention with the mediating effect of trust in online communities (evidence from skincare consumers on facebook). In: Ruhuna Uo, editor. 14th International Conference in Management and Economics. University of Ruhuna: Faculty of Management and Finance; 2025. p. 457–67.

[26] Agus P, Anwar S, Fahmi K. The role of buzz and viral marketing on SMEs online shop marketing performance: CB-SEM AMOS analysis. IJOSMAS. 2023;4(3):1–7.

[27] Ghosh P, Upadhyay S, Srivastava V, Dhiman R, Yu L. How influencer characteristics drive Gen Z behavioural intentions of selecting fast-food restaurants: mediating roles of consumer emotions and self-construal. BFJ. 2024;126(12):4072–92. doi: 10.1108/bfj-12-2023-1154

[28] Shaheen R, Ghasemi M. Make it real with gen Z! The impact AR reality congruence on brand information sharing: exploring a sequential mediation mechanism. Int J Data Netw Sci. 2025;9(1):227–42. doi: 10.5267/j.ijdns.2024.8.001

[29] Mahajan AI, Taggar R, Gupta S. Augmented reality in E-Commerce: a dual value and barrier-based perspective on Gen Z’s satisfaction and AR-Integrated shopping behavior. J Retail Consum Serv. 2025;87:104417. doi: 10.1016/j.jretconser.2025.104417

[30] Anwar RS, Ahmed RR, Streimikiene D, Strielkowski W, Streimikis J. Customer engagement, innovation, and sustainable consumption: analyzing personalized, innovative, sustainable phygital products. J Innov Knowl. 2025;10(1):100642. doi: 10.1016/j.jik.2024.100642

[31] Davis FD. Perceived usefulness, perceived ease of use, and user acceptance of information technology. MIS Q. 1989;13(3):319–40. doi: 10.2307/249008

[32] Davis FD. User acceptance of information technology: system characteristics, user perceptions and behavioral impacts. Int J Man-Mach Stud. 1993;38(3):475–87. doi: 10.1006/imms.1993.1022

[33] Venkatesh V, Davis FD. A theoretical extension of the technology acceptance model: four longitudinal field studies. Manag Sci. 2000;46(2):186–204. doi: 10.1287/mnsc.46.2.186.11926

[34] Venkatesh V, Bala H. Technology acceptance model 3 and a research agenda on interventions. Decis Sci. 2008;39(2):273–315. doi: 10.1111/j.1540-5915.2008.00192.x

[35] Fahmi MA, Kostini N, Sunaryo Putra WBT. Exploring hybrid learning readiness and acceptance model using the extended TAM 3 and TPB approach: an empirical analysis. IJRBS. 2022;11(8):321–34. doi: 10.20525/ijrbs.v11i8.2144

[36] Vargo SL, Lusch RF. Evolving to a new dominant logic for marketing. J Mark. 2004;68(1):1–17. doi: 10.1509/jmkg.68.1.1.24036

[37] Batat W. Experiential marketing: consumer behavior, customer experience and the 7Es. 1st ed. Abingdon, Oxon; New York, NY: Routledge; 2019.

[38] Grönroos C. Value co-creation in service logic: a critical analysis. Mark Theory. 2011;11(3):279–301. doi: 10.1177/1470593111408177

[39] Pangarkar A, Shukla P. Conspicuous and inconspicuous consumption of luxury goods in a digital world: insights, implications, and future research directions. Int J Advert. 2023;42(7):1226–38. doi: 10.1080/02650487.2023.2246260

[40] Witell L. Service innovation and management. Cham: Springer; 2025.

[41] Banik S, Gao Y. Exploring the hedonic factors affecting customer experiences in phygital retailing. J Retail Consum Serv. 2023;70:103147. doi: 10.1016/j.jretconser.2022.103147

[42] Banik S. Exploring the involvement-patronage link in the phygital retail experiences. J Retail Consum Serv. 2021;63:2–12. doi: 10.1016/j.jretconser.2021.102739

[43] Belghiti S, Ochs A, Lemoine J-F, Badot O. The phygital shopping experience: an attempt at conceptualization and empirical investigation. In: Rossi P, Krey N, editors. AMSWMC 2017 (Academy of Marketing Science World Marketing Congress, 2017). Cham: Springer; 2017.

[44] Alesanco-Llorente M, Reinares-Lara E, Pelegrín-Borondo J, Olarte-Pascual C. Mobile-assisted showrooming behavior and the (r)evolution of retail: the moderating effect of gender on the adoption of mobile augmented reality. Technol Forecast Soc Change. 2023;191:122514. doi: 10.1016/j.techfore.2023.122514

[45] Yi J, Oh YK, Kim J-M. Unveiling the drivers of satisfaction in mobile trading: Contextual mining of retail investor experience through BERTopic and generative AI. J Retail Consum Serv. 2025;82:104066. doi: 10.1016/j.jretconser.2024.104066

[46] Marbach J, Lages C, Nunan D, Ekinci Y. Consumer engagement in online brand communities: the moderating role of personal values. EJM. 2019;53(9):1671–700. doi: 10.1108/ejm-10-2017-0721

[47] Akram MF, Ali MR, Kadirova Z. Role of consumer digital literacy in consumer behavioral intentions: Mediating effect of perceived ease of use, usefulness and customer engagement. J Educ Res. 2023;13(1):105–30.

[48] Angkhasakulkiat R, Puriwat W, Hoonsopon D. Digital literacy for business performance: a study of entrepreneurs. HighTech Innov J. 2025;6(1):273–88. doi: 10.28991/hij-2025-06-01-018

[49] Semente Efigenia Madalena Mario, Whyte Grafton. Assessing Digital Literacy Among Namibian Millennials and Impact on Comsumer Decision-Making Styles: A Case Study. International Journal of Applued Management Sciences and Engineering (IJAMSE). 2020;7(1):54–73 10.4018/ijamse.2020010103

[50] Sharma K, Trott S, Sahadev S, Singh R. Emotions and consumer behaviour: a review and research agenda. Int J Consum Stud. 2023;47(6):2396–416. doi: 10.1111/ijcs.12937

[51] Nuralam IP, Yudiono N, Fahmi MRA, Yuliaji ES, Hidayat T. Perceived ease of use, perceived usefulness, and customer satisfaction as driving factors on repurchase intention: the perspective of the e-commerce market in Indonesia. Cogent Bus Manag. 2024;11(1). doi: 10.1080/23311975.2024.2413376

[52] Musa HG, Fatmawati I, Nuryakin N, Suyanto M. Marketing research trends using technology acceptance model (TAM): a comprehensive review of researches (2002–2022). Cogent Bus Manag. 2024;11(1):2329375. doi: 10.1080/23311975.2024.2329375

[53] Nagaraj S. Marketing analytics for customer engagement: a viewpoint. Int J Inf Syst Soc Change. 2020;11(2):1–15. doi: 10.4018/IJISSC.2020040104

[54] Bowden JL-H. The process of customer engagement: a conceptual framework. J Mark Theory Pract. 2009;17(1):63–74. doi: 10.2753/mtp1069-6679170105

[55] Xu FZ, Wang Y. Enhancing employee innovation through customer engagement: The role of customer interactivity, employee affect, and motivations. J Hosp Tour Res. 2019;44(2):351–76. doi: 10.1177/1096348019893043

[56] Binsaeed RH, Yousaf Z, Grigorescu A, Chitescu RI, Nassani AA, Samoila A. Customer engagement and customer relationship management capabilities’ effects on innovation performance and customer distrust’s moderating role. Sustainability. 2023;15(12):9475. doi: 10.3390/su15129475

[57] Bosisio J. A research landscape on customer co-creation value: a systematic literature network analysis. Ital J Mark. 2024;2024(3):339–68. doi: 10.1007/s43039-024-00092-9

[58] Siu NYM, Zhang TJ, Yeung RS-P. The bright and dark sides of online customer engagement on brand love. JCM. 2023;40(7):957–70. doi: 10.1108/jcm-01-2022-5118

[59] Lusch RF, Vargo SL. Service-dominant logic: premises, perspectives, possibilities. Cambridge: Cambridge University Press; 2014.

[60] Storbacka K, Brodie RJ, Böhmann T, Maglio PP, Nenonen S. Actor engagement as a microfoundation for value co-creation. J Bus Res. 2016;69(8):3008–17. doi: 10.1016/j.jbusres.2016.02.034

[61] Bridgenext TT. Bridgenext; 2024. [cited 2025 Sept 17]. Available from: https://www.bridgenext.com/blog/mapping-customer-experience-journey-with-phygital-blend/

[62] Stremersch S, Cabooter E, Guitart IA, Camacho N. Customer insights for innovation: a framework and research agenda for marketing. J Acad Mark Sci. 2024;53(1):29–51. doi: 10.1007/s11747-024-01051-8

[63] Schaarschmidt M, Walsh G, Evanschitzky H. Customer interaction and innovation in hybrid offerings: Investigating moderation and mediation effects for goods and services innovation. J Serv Res. 2017;21(1):119–34. doi: 10.1177/1094670517711586

[64] Nguyen HS. The impact of value co-creation behavior on customer loyalty in the service domain. Heliyon. 2024;10(9):e30278. doi: 10.1016/j.heliyon.2024.e30278 38694059 PMC11061728

[65] Amer SM. The impact of customer perceived innovativeness on customer happiness and revisit intention through customer value co-creation behaviors in quick-service restaurants. IJIRSS. 2025;8(2):2296–309. doi: 10.53894/ijirss.v8i2.5683

[66] Bouchriha Z, Farid S, Ouiddad S. Enhancing value co-creation behaviors through customer engagement in the Moroccan hotel context: how does it influence customer satisfaction and brand image? J Qual Assur Hosp Tour. 2023;25(6):1581–606. doi: 10.1080/1528008X.2023.2165595

[67] Edvardsson B, Tronvoll B. A new conceptualization of service innovation grounded in S‐D logic and service systems. Int J Qual Serv Sci. 2013;5(1):19–31. doi: 10.1108/17566691311316220

[68] Skålén P, Aal KA, Edvardsson B. Cocreating the arab spring: understanding transformation of service systems in contention. J Serv Res. 2015;18(3):250–64. doi: 10.1177/1094670514559700

[69] Baehaqi M, Rizana D, Cahyono RS, Naryananda HPB. Co - creating experience in phygital platform: exploration of customer experience and patronage intention in retail business. IJEBE. 2024;13(3):457–76. doi: 10.26418/jebik.v13i3.81166

[70] Muniz F, Guzmán F. The impact of brand value co-creation on perceived CSR authenticity and brand equity. JPBM. 2023;32(8):1338–54. doi: 10.1108/jpbm-02-2023-4340

[71] Oklevik O, Nysveen H, Pedersen PE. Exploring the relationship between co-creation (DART), brand experience strength, and brand satisfaction: a brand engagement perspective. J Mark Theory Pract. 2022;32(1):1–24. doi: 10.1080/10696679.2022.2120013

[72] Royo-Vela M, Frau M, Ferrer A. The role of value co-creation in building trust and reputation in the digital banking era. Cogent Bus Manag. 2024;11(1):1–19. doi: 10.1080/23311975.2024.2375405

[73] Grönroos C, Voima P. Critical service logic: making sense of value creation and co-creation. J Acad Mark Sci. 2012;41(2):133–50. doi: 10.1007/s11747-012-0308-3

[74] Chapman A, Dilmperi A. Luxury brand value co-creation with online brand communities in the service encounter. J Bus Res. 2022;144:902–21. doi: 10.1016/j.jbusres.2022.01.068

[75] Basile V, Brandão A, Ferreira M. Does user-generated content influence value co-creation in the context of luxury fashion brand communities? Matching inclusivity and exclusivity. Ital J Mark. 2024;2024(4):419–44. doi: 10.1007/s43039-024-00103-9

[76] Stan V, Baltas G, Pourot-Feenstra F. Self-service technologies in retail stores: how phygital retailing creates customer value and drives choice confidence. Inf Technol People. 2024;39(1):354–74. doi: 10.1108/itp-09-2022-0678

[77] Alexander B, Varley R. Retail futures: customer experience, phygital retailing, and the Experiential Retail Territories perspective. J Retail Consum Serv. 2025;82:104108. doi: 10.1016/j.jretconser.2024.104108

[78] Jianhua C, Ali MH, Said RM. Perceived values, customer engagement, and collective efficacy with C-A-C on group buying app’s purchase intention. IJARBSS. 2024;14(9). doi: 10.6007/ijarbss/v14-i9/22717

[79] Bapat D, Hollebeek LD. Customer value, customer engagement, and customer-based brand equity in the context of a digital payment app. MIP. 2023;41(7):837–53. doi: 10.1108/mip-09-2022-0417

[80] Wang Y. The Impact of Customer Engagement on Perceived Value in the Context of E-commerce Livestreaming. Journal of Distribution Science. 2024;22(2):51–61. 10.15722/jds.22.02.202402.51

[81] Guzzetti A, Crespi R, Belvedere V. Phygital luxury experiences. A correspondence analysis on retail technologies. International Journal of Consumer Studies. 2024;48(2):1–15. doi: 10.1111/ijcs.13008

[82] Alexander MJ, Jaakkola E, Hollebeek LD. Zooming out: actor engagement beyond the dyadic. J Serv Manag. 2018;29(3):333–51. doi: 10.1108/josm-08-2016-0237

[83] Tedja B, Al Musadieq M, Kusumawati A, Yulianto E. Systematic literature review using PRISMA: exploring the influence of service quality and perceived value on satisfaction and intention to continue relationship. Futur Bus J. 2024;10(1):1–39. doi: 10.1186/s43093-024-00326-4

[84] Yum K, Kim J. The influence of perceived value, customer satisfaction, and trust on loyalty in entertainment platforms. Appl Sci. 2024;14(13):5763. doi: 10.3390/app14135763

[85] Blut M, Chaney D, Lunardo R, Mencarelli R, Grewal D. Customer perceived value: a comprehensive meta-analysis. J Serv Res. 2023;27(4):501–24. doi: 10.1177/10946705231222295

[86] Nguyen TKQ. E-banking service quality and customer satisfaction with moderator factor. Emerg Sci J. 2025;9(2):725–40. doi: 10.28991/ESJ-2025-09-02-012

[87] Mofokeng TE, Tan AWK. The impact of online shopping attributes on customer satisfaction and loyalty: Moderating effects of e-commerce experience. Cogent Business & Management. 2021;8(1):1–33. doi: 10.1080/23311975.2021.1968206

[88] Umboh SFBW, Tulung JE, Wangke SJC. The influence of perceived value to customer loyalty with customer satisfaction as an intervening variable on ESSE Brand Users in Manado. RAMP. 2024;2(1):1–19. doi: 10.58784/ramp.89

[89] Wang L, Li L, Chen Y. Research on the influence mechanism of perceived customer service quality on consumers’ purchase decisions on e-commerce platforms. In: Tu YP, Chi M, editors. WHICEB 2024. Cham: Springer; 2024.

[90] Mittal V, Han K, Frennea C, Blut M, Shaik M, Bosukonda N, et al. Customer satisfaction, loyalty behaviors, and firm financial performance: what 40 years of research tells us. Mark Lett. 2023;34(2):171–87. doi: 10.1007/s11002-023-09671-w

[91] Singh P, Singh V. The power of AI: enhancing customer loyalty through satisfaction and efficiency. Cogent Bus Manag. 2024;11(1):1–14. doi: 10.1080/23311975.2024.2326107

[92] Yum K, Yoo B. The impact of service quality on customer loyalty through customer satisfaction in mobile social media. Sustainability. 2023;15(14):11214. doi: 10.3390/su151411214

[93] Khowjoy K, Sriplang N, Kaewsrem S, Phakamach V, Petmee DP, Chayomchri DA. A Study of the Important Factors Influencing Customer Loyalty: A Literature Review. Journal of Business and Management. 2022;24(12):16–21. 10.9790/487X-2412031621

[94] Mishra S, Malhotra G, Arora V, Mukhopadhyay S. Omnichannel retailing: does it empower consumers and influence patronage?. International Journal of Retail & Distribution Management. 2021;50(2):229–50. doi: 10.1108/IJRDM-04-2021-0199

[95] Kondybayeva S, Daribayeva M, Fiume R, Abilda S, Staroverova O, Ponkratov V, et al. A new concept of transforming service: impact of generative voice chatbots on customer satisfaction and banking industry productivity. Emerg Sci J. 2024;8(6):2278–311. doi: 10.28991/esj-2024-08-06-09

[96] Jo H, Ahn HY. Understanding digital engagement: factors influencing awareness and satisfaction of digital transformation. Discov Comput. 2024;27(1). doi: 10.1007/s10791-024-09455-4

[97] Ahmad F, Mustafa K, Hamid SAR, Khawaja KF, Zada S, Jamil S, et al. Online customer experience leads to loyalty via customer engagement: moderating role of value co-creation. Front Psychol. 2022;13. doi: 10.3389/fpsyg.2022.897851 PMC9369676 35967631

[98] Shoukat MH, Ramkissoon H. Customer delight, engagement, experience, value co-creation, place identity, and revisit intention: a new conceptual framework. J Hosp Mark Manag. 2022;31(6):757–75. doi: 10.1080/19368623.2022.2062692

[99] Jiang K, Zheng J, Qin M, Luo S. Perceived retailer innovativeness and its impact on customer engagement behavior in smart retailing. Serv Bus. 2024;18(2):255–85. doi: 10.1007/s11628-024-00560-4

[100] Brodie RJ, Hollebeek LD, Jurić B, Ilić A. Customer engagement: conceptual domain, fundamental propositions, and implications for research. J Serv Res. 2011;14(3):252–71. doi: 10.1177/1094670511411703

[101] Pantano E, Gandini A. Exploring the forms of sociality mediated by innovative technologies in retail settings. Comput Hum Behav. 2017;77:367–73. doi: 10.1016/j.chb.2017.02.036

[102] Lemon KN, Verhoef PC. Understanding customer experience throughout the customer journey. J Mark. 2016;80(6):69–96. doi: 10.1509/jm.15.0420

[103] Pansari A, Kumar V. Customer engagement: the construct, antecedents, and consequences. J Acad Mark Sci. 2016;45(3):294–311. doi: 10.1007/s11747-016-0485-6

[104] Zhou Y, Kumar S, Furuoka F. Enhancing customer value co-creation and stickiness in social commerce: integrating PLS-SEM and NCA for deeper insights into customer-to-customer dynamics. Humanit Soc Sci Commun. 2024;11(1):46. doi: 10.1057/s41599-023-02586-x

[105] Rasaputhra S, Peiris V, Magallagoda R, Panditasekara C, Wisenthige K, Jayasuriya N. Do technological, environmental and entrepreneurial factors affect social commerce adoption? JSBED. 2024;31(4):764–85. doi: 10.1108/jsbed-09-2023-0420

[106] Winell E, Nilsson J, Lundberg E. Customer engagement behaviors on physical and virtual engagement platforms. JSM. 2023;37(10):35–50. doi: 10.1108/jsm-03-2023-0084

[107] Kahawandala N, Peter S, Niwunhella H. Profiling purchasing behavior of Generation Z. 2020 International Research Conference on Smart Computing and Systems Engineering (SCSE); 2020. p. 155–60. 10.1109/scse49731.2020.9313038

[108] Saunders MNK, Lewis P, Thronhill A. Research methods for business students. Pearson Education Limited; 2023.

[109] Ranjith P, Nisansala S, Jayasingha N, Weerasekara K, Wisenthige K, Dayapathirana N. Does social media information credibility influence social commerce purchase intention of skincare products? Evidence from Facebook. PLoS One. 2025;20(10):e0334126. doi: 10.1371/journal.pone.0334126 41124229 PMC12543200

[110] Roy SK, Singh G, Sadeque S, Harrigan P, Coussement K. Customer engagement with digitalized interactive platforms in retailing. J Bus Res. 2023;164:114001. doi: 10.1016/j.jbusres.2023.114001

[111] Pires PB, Prisco M, Delgado C, Santos JD. A conceptual approach to understanding the customer experience in e-commerce: an empirical study. JTAER. 2024;19(3):1943–83. doi: 10.3390/jtaer19030096

[112] Wardhani D, Hesti S, Dwityas NA. Digital literacy: a survey level digital literacy competence among university students in Jakarta. IJELS. 2019;4(4):1131–8. doi: 10.22161/ijels.4434

[113] Guerola-Navarro V, Stratu-Strelet D, Botella-Carrubi D, Gil-Gomez H. Media or information literacy as variables for citizen participation in public decision-making? A bibliometric overview. Sustain Technol Entrep. 2023;2(1):100030. doi: 10.1016/j.stae.2022.100030

[114] Lim WM, Rasul T. Customer engagement and social media: revisiting the past to inform the future. J Bus Res. 2022;148:325–42. doi: 10.1016/j.jbusres.2022.04.068

[115] Chen X, Zhang G. Investigating the impact of customer engagement on customer innovation behaviors in online brand communities. JEMS. 2022;5(1):p1. doi: 10.30560/jems.v5n1p1

[116] Yaşlıoğlu M, Çalışkan BÖÖ, Şap Ö. The role of innovation and perceived service quality in creating customer value: a study on employees of a call center establishment. Procedia Soc Behav Sci. 2013;99:629–35. doi: 10.1016/j.sbspro.2013.10.533

[117] Malik MI, Ahsan R. Towards innovation, co-creation and customers’ satisfaction: a banking sector perspective. APJIE. 2019;13(3):311–25. doi: 10.1108/apjie-01-2019-0001

[118] Galvagno M, Dalli D. Theory of value co-creation: a systematic literature review. Manag Serv Qual. 2014;24(6):643–83. doi: 10.1108/msq-09-2013-0187

[119] Ranjan KR, Read S. Value co-creation: concept and measurement. J Acad Mark Sci. 2014;44(3):290–315. doi: 10.1007/s11747-014-0397-2

[120] Croitoru G, Capatina A, Florea NV, Codignola F, Sokolic D. A cross-cultural analysis of perceived value and customer loyalty in restaurants. Eur Res Manag Bus Econ. 2024;30(3):100265. doi: 10.1016/j.iedeen.2024.100265

[121] Maghembe M, Magasi C. The role of customer perceived value, brand trust and service personalization in shaping customer loyalty. Int J Manag Account Econ. 2024;11(9):1197–219. doi: 10.5281/zenodo.13761101

[122] Šapić S, Topalović S, Marinković V. The influence of perceived value dimensions on customer loyalty. Econ Themes. 2014;52(4):392–408. doi: 10.1515/ethemes-2014-0025

[123] Gálvez-Ruiz P, Calabuig F, Grimaldi-Puyana M, González-Serrano MH, García-Fernández J. The effect of perceived quality and customer engagement on the loyalty of users of Spanish fitness centres. ARLA. 2023;36(4):445–62. doi: 10.1108/arla-01-2023-0014

[124] Erciş A, Ünal S, Candan FB, Yıldırım H. The effect of brand satisfaction, trust and brand commitment on loyalty and repurchase intentions. Procedia Soc Behav Sci. 2012;58:1395–404. doi: 10.1016/j.sbspro.2012.09.1124

[125] Nguyen TLH. Fostering loyalty as repurchase intention: the role of relationship marketing and word-of-mouth. Int J Health Econ Policy. 2021;6(2):72–8. doi: 10.11648/j.hep.20210602.16

[126] Hair JF, Risher JJ, Sarstedt M, Ringle CM. When to use and how to report the results of PLS-SEM. EBR. 2019;31(1):2–24. doi: 10.1108/ebr-11-2018-0203

[127] Wijesinghe U, Mapitiyage V, Wickramarathne C, Wickramage C, Wisenthige K, Aluthwala C. Does management support drive sustained agile usage? a serial mediation model and cIPMA perspective. PLoS One. 2025;20(2):e0316538. doi: 10.1371/journal.pone.0316538 39908259 PMC11798488

[128] Hair JF, Hult GTM, Ringle CM, Sarstedt M. A Primer on Partial Least Squares Structural Equation Modeling (PLS-SEM). 2nd ed. Thousand Oaks (CA): Sage; 2017.

[129] Chin WW. The partial least squares approach to structural equation modeling. In: Marcoulides GA, editor. Modern methods for business research. Mahwah (NJ): Lawrence Erlbaum Associates; 1998. p. 295–336.

[130] Hair JF, Hult GTM, Ringle CM, Sarstedt M, Danks NP, Ray S. Partial least squares structural equation modeling (PLS-SEM) using R: a workbook. Cham: Springer; 2021.

[131] Nunnally JC, Bernstein IH. Psychometric theory. 3rd ed. New York: McGraw-Hill; 1994.

[132] Zainol Z, Habidin NF, Osman J, Yahaya R. The key qualities of a strong customer engagement in the customer-brand relationship context. IJARBSS. 2016;6(12). doi: 10.6007/ijarbss/v6-i12/2487

[133] Fornell C, Larcker DF. Evaluating structural equation models with unobservable variables and measurement error. J Mark Res. 1981;18(1):39. doi: 10.2307/3151312

[134] Diamantopoulos A, Siguaw JA. Formative versus reflective indicators in organizational measure development: a comparison and empirical illustration. Br J Manag. 2006;17(4):263–82. doi: 10.1111/j.1467-8551.2006.00500.x

[135] Hu L, Bentler PM. Cutoff criteria for fit indexes in covariance structure analysis: conventional criteria versus new alternatives. Struct Equ Modeling. 1999;6(1):1–55. doi: 10.1080/10705519909540118

[136] Henseler J, Hubona G, Ray PA. Using PLS path modeling in new technology research: updated guidelines. Ind Manag Data Syst. 2016;116(1):2–20. doi: 10.1108/imds-09-2015-0382

[137] Pizzi G, Scarpi D, Pantano E. Artificial intelligence and the new forms of interaction: Who has the control when interacting with a chatbot? J Bus Res. 2021;129:878–90. doi: 10.1016/j.jbusres.2020.11.006

[138] Grewal D, Roggeveen AL, Nordfält J. The future of retailing. J Retail. 2017;93(1):1–6. doi: 10.1016/j.jretai.2016.12.008

[139] Gustafsson A, Öberg C, Shams P. Enhancing the phygital customer experience in the digital world. In: Witell L, editor. Service innovation and management: digitalization, service infusion and customer experience. Cham: Springer Nature Switzerland; 2025. p. 113–26.

[140] Pantano E, Timmermans H. What is known about retail experience and which research methods to use? J Retail Consum Serv. 2014;21(5):709–11.

[141] Roper S, Bourke J. Innovating into trouble: when innovation leads to customer complaints. Res Policy. 2022;51(10):104593. doi: 10.1016/j.respol.2022.104593

[142] Sawhney M, Verona G, Prandelli E. Collaborating to create: the Internet as a platform for customer engagement in product innovation. J Interact Mark. 2005;19(4):4–17. doi: 10.1002/dir.20046

[143] Giang HTT, Dung LT, Tien HT, Nhu CTB. Customer empowerment and engagement on sharing platform in the retailing sector: testing the mediating effects of service innovation and platform trust. J Innov Entrep. 2024;13(1). doi: 10.1186/s13731-024-00431-2

[144] Heidenreich S, Jordanow S, Kraemer T, Obschonka M. Together forever? How customer co‐creation affects the adoption of digital service innovations over time. J Product Innov Manag. 2024;41(5):1062–90. doi: 10.1111/jpim.12727

[145] Nohutlu ZD, Englis BG, Groen AJ, Constantinides E. Innovating with the customer: co-creation motives in online communities. Int J Electron Commer. 2023;27(4):523–57. doi: 10.1080/10864415.2023.2255111

[146] Yi X, Ul Haq J, Ahmed S. Impact of customer participation in value co-creation on customer wellbeing: A moderating role of service climate. Frontiers in Psychology. 2023;13:null. doi: 10.3389/fpsyg.2022.877083 PMC9887321 36733868

[147] Solakis K, Peña-Vinces J, Lopez-Bonilla JM. Value co-creation and perceived value: a customer perspective in the hospitality context. Eur Res Manag Bus Econ. 2022;28(1):100175. doi: 10.1016/j.iedeen.2021.100175

[148] Li Y. The impact of customer perceived value in the contemporary art distribution chain. RGSA. 2024;18(7):e05670. doi: 10.24857/rgsa.v18n7-083

[149] Zeithaml VA. Consumer perceptions of price, quality, and value: a means-end model and synthesis of evidence. J Mark. 1988;52(3):2–22. doi: 10.1177/002224298805200302

[150] Sebastián-Morillas A, Monfort A, López-Vázquez B. Effects of perceived value and customer service on brand satisfaction. J Promot Manag. 2023;30(2):187–203. doi: 10.1080/10496491.2023.2253231

[151] Le BN, Nguyen N. The impact of perceived value on consumers’ positive word-of-mouth intention toward energy-efficient appliances. IJEEP. 2024;14(2):383–93. doi: 10.32479/ijeep.15655

[152] Khawaja L, Ali AA, Mostapha N. The mediating effect of customer satisfaction in relationship with service quality, corporate social responsibility, perceived quality and brand loyalty. Manag Sci Lett. 2021;11:763–72. doi: 10.5267/j.msl.2020.10.030

[153] Ramaswamy V, Ozcan K. Brand value co-creation in a digitalized world: an integrative framework and research implications. Int J Res Mark. 2016;33(1):93–106. doi: 10.1016/j.ijresmar.2015.07.001

[154] Oliver RL. A cognitive model of the antecedents and consequences of satisfaction decisions. J Mark Res. 1980;17(4):460. doi: 10.2307/3150499

[155] Verhoef PC, Kannan PK, Inman JJ. From multi-channel retailing to omni-channel retailing: Introduction to the special issue on multi-channel retailing. J Retail. 2015;91(2):174–81. doi: 10.1016/j.jretai.2015.02.005

[156] Helsper EJ. A corresponding fields model for the links between social and digital exclusion. Commun Theor. 2012;22(4):403–26. doi: 10.1111/j.1468-2885.2012.01416.x

[157] Calder BJ, Malthouse EC, Schaedel U. An experimental study of the relationship between online engagement and advertising effectiveness. J Interact Mark. 2009;23(4):321–31. doi: 10.1016/j.intmar.2009.07.002

[158] Yadav MS, Pavlou PA. Technology-enabled interactions in digital environments:a conceptual foundation for current and future research. J Acad Mark Sci. 2019;48(1):132–6. doi: 10.1007/s11747-019-00712-3

[159] Wedel M, Bigné E, Zhang J. Virtual and augmented reality: advancing research in consumer marketing. Int J Res Mark. 2020;37(3):443–65. doi: 10.1016/j.ijresmar.2020.04.004

[160] Shankar V, Narang U. Emerging market innovations: unique and differential drivers, practitioner implications, and research agenda. J Acad Mark Sci. 2019;48(5):1030–52. doi: 10.1007/s11747-019-00685-3

[161] Rather RA. Demystifying the effects of perceived risk and fear on customer engagement, co-creation and revisit intention during COVID-19: a protection motivation theory approach. J Destin Mark Manag. 2021;20:100564. doi: 10.1016/j.jdmm.2021.100564

[162] Hapsari R, Clemes MD, Dean D. The role of customer engagement in enhancing passenger loyalty in IndonesianAirline industry: relationship marketing approach. APMBA. 2015;3(3):135–44. doi: 10.21776/ub.apmba.2015.003.03.1

[163] Hoyer WD, Kroschke M, Schmitt B, Kraume K, Shankar V. Transforming the customer experience through new technologies. J Interact Mark. 2020;51(1):57–71. doi: 10.1016/j.intmar.2020.04.001

[164] Prastiwi EH, Surachman S, Hussein AS, editors. The role of value co-creation in improving customer loyalty with customer satisfaction as mediating variable. Atlantis Press; 2019.

[165] Mishra S, Malhotra G, Chatterjee R, Shukla Y. Consumer retention through phygital experience in omnichannel retailing: role of consumer empowerment and satisfaction. J Strateg Mark. 2021;31(4):749–66. doi: 10.1080/0965254x.2021.1985594

