# Supplementary material for: Unlocking Gen Z engagement in phygital retailing: Evidence from a multi-mediated customer experience model
Source: PLoS One. 2026 Aug 6;21(8):e0353490. doi: 10.1371/journal.pone.0353490 (PMC13446700; doi:10.1371/journal.pone.0353490)
Supplement: Supplementary file 1 [file pone.0353490.s002.docx]

**Academic Editor’s Comments**

| **Editor’s Comment** | **Response to the Comment** |
| --- | --- |
| 1. Please ensure that your manuscript meets PLOS ONE's style requirements, including those for file naming. | Thank you for your valuable insight. We acknowledge that the attached files were able to meet PLOS ONE’s style requirements. The files’ names that we attached as follows,   1. Final PLOS ONE manuscript 2. Supplementary documents    2.1 Author Contributions    2.2 Author Summary    2.3 PLOS ONE cover Letter    2.4 Title Page    2.5 Fig1 (Literature Search Flow Diagram)    2.6 Fig2 (Conceptual Framework)    2.7 Fig3 (Measurement Model) |
| 2. Thank you for submitting the above manuscript to PLOS ONE. During our internal evaluation of the manuscript, we found significant text overlap between your submission and previous work in the [introduction, conclusion, etc.].  We would like to make you aware that copying extracts from previous publications, especially outside the methods section, word-for-word is unacceptable. In addition, the reproduction of text from published reports has implications for the copyright that may apply to the publications.  Please revise the manuscript to rephrase the duplicated text, cite your sources, and provide details as to how the current manuscript advances on previous work. Please note that further consideration is dependent on the submission of a manuscript that addresses these concerns about the overlap in text with published work. | Thank you for your valuable feedback and for highlighting the concerns regarding text overlap in our manuscript.  We would like to clarify that prior to submission, the manuscript was evaluated using Turnitin web application to ensure originality of the study, and professional proofreading was also performed to enhance clarity of the writing and language quality. We acknowledge that certain sections, particularly the introduction and conclusion, major changes included in text due to overlap previous work.  In response to your comments, we have carefully revised the manuscript to ensure that all previously overlapping content has been corrected using our own words. Appropriate citations have been retained and strengthened where necessary. Additionally, we also included reviewers’ recommended citation into the manuscript. Not only that we have further emphasized the novel contributions of this study to clearly demonstrate how it advances beyond existing literature and a major theoretical clarity were added to the manuscript.  We sincerely appreciate your guidance, which has helped us to improve the quality and originality of our manuscript into journal accepted standard. We hope that the revised manuscript now adequately addresses the comments raised and meets the journal’s requirements for further consideration. |
| 3. Your ethics statement should only appear in the Methods section of your manuscript. If your ethics statement is written in any section besides the Methods, please delete it from any other section. | Thank you for your valuable comment regarding the replacement of the ethics statement.  We confirm that the ethics statement has been included only within the methods section of the manuscript. In our submission, this section was titled “Data and Methodology”. To improve the clarity and align with the journal’s formatting expectations, we have revised the section heading to “Methods” and ensured that the ethics statement appears exclusively within this section and not elsewhere in the manuscript except in the title page due to journal requirement. Although, journals require exclude it from the title page. We appreciate your comment and have made the necessary adjustment accordingly. |
| 4. In this instance it seems there may be acceptable restrictions in place that prevent the public sharing of your minimal data. However, in line with our goal of ensuring long-term data availability to all interested researchers, PLOS’ Data Policy states that authors cannot be the sole named individuals responsible for ensuring data access. Data requests to a non-author institutional point of contact, such as a data access or ethics committee, helps guarantee long term stability and availability of data. Providing interested researchers with a durable point of contact ensures data will be accessible even if an author changes email addresses, institutions, or becomes unavailable to answer requests.  Before we proceed with your manuscript, please also provide non-author contact information (phone/email/hyperlink) for a data access committee, ethics committee, or other institutional body to which data requests may be sent. If no institutional body is available to respond to requests for your minimal data, please consider if there any institutional representatives who did not collaborate in the study, and are not listed as authors on the manuscript, who would be able to hold the data and respond to external requests for data access? If so, please provide their contact information (i.e., email address). Please also provide details on how you will ensure persistent or long-term data storage and availability. | We thank the editorial team for the valuable guidance regarding data accessibility and long-term availability.  In accordance with the PLOS Data Policy, we confirm that a non-author institutional point of contact has been designated to ensure sustained accessibility of the research data. The data are securely stored in the institutional repository of SLIIT Business School, which is overseen by the Ethics Committee.  The designated non-author contact is:   - Prof. Wasantha Rajapaksha - Head – Ethics Committee / Institutional Repository, SLIIT Business School - Institutional Profile: <https://www.sliit.lk/academic/academic-staff/wasantha.r> - Contact: +94 71 017 4943 - Data Repository Link: [SLIITERCSBS202512](https://mysliit-my.sharepoint.com/:f:/g/personal/nirmani_d_sliit_lk/IgDTewzq8AwgSbknmc-F_ln4AUdS4nrfehdMa6W8CY96vsI?e=dhhN3b)   Researchers requesting access to the dataset may contact the above institutional representative for data access inquiries and permissions.  Regarding long-term preservation, the dataset is maintained within the SLIIT institutional repository, which is managed under university-level governance policies ensuring secure archival storage, routine backups, and continued availability independent of individual authors. This structure ensures that data access will remain stable even in cases of author relocation or unavailability.  We trust this arrangement satisfies the journal’s data accessibility requirements. |
| 5. If the reviewer comments include a recommendation to cite specific previously published works, please review and evaluate these publications to determine whether they are relevant and should be cited. There is no requirement to cite these works unless the editor has indicated otherwise. | We thank the reviewer for suggesting these relevant studies. We carefully evaluated the recommended publications and found them to be pertinent to our research. Accordingly, we have incorporated these references in the revised manuscript to strengthen the context of our study. |

**Reviewers’ Comments**

| **1^st^ Reviewers’ Comment** | **Response to the Comment** |
| --- | --- |
| 1. The study focuses on Sri Lanka’s consumer electronics retail sector, which is valuable for emerging market insights. However, the manuscript could better justify why this context is particularly suitable for studying phygital experiences and Gen Z engagement. | We thank the reviewer for this valuable comment. In response to that, lines 161 - 170 have been added and changed to provide an explanation on why consumer electronics retail sector is suitable for studying phygital experiences and Gen Z engagement. *“products such as smartphones, televisions, etc., which typically involve a combination of online information search and in-store evaluation prior to purchase. This blend of experience perfectly aligns with the concept of phygital, which integrates physical and digital touchpoints to enhance customer experience [20, 21]. According to [22], the country (Sri Lanka) demonstrates a high level of digital readiness, amounting to over 12 million internet users, 29 million cellular mobile subscriptions and 21 million mobile broadband subscriptions. Hence, the widespread mobile connectivity suggests that consumers are routinely exposed to both online and offline touchpoints, enabling them to seamlessly interact with the mixed realms . Furthermore, [17] has stated that,”*  Next, the lines from 179 – 187, explains why this context is particularly suitable for studying phygital experiences and Gen Z engagement.  *“Additionally, studies by [28, 29] highlight that AR enhances the shopping experience of Gen Z by allowing them to visualize products in real-time and interact with virtual components. Retailers, who integrate AR technology into their business operations, such as virtual try-ons or interactive product demos can offer seamless experiences that fascinate Gen Z consumers to sustain their loyalty to the retailers. Aligning with this, the consumer electronics category is especially suitable because it includes products such as mobile devices, computers, and televisions, which commonly require online search, comparison, and in-store inspection before making the purchase. Therefore, it naturally reflects the phygital consumption patterns.”* |
| 2. The concept of phygital experience is introduced well, but the manuscript could benefit from a clearer operational definition. How do the authors distinguish phygital experience from omnichannel or multichannel retailing? | We are grateful for this valuable suggestion. In response, lines 135 - 147 have been added to provide an explanation on how to distinguish phygital experience from omnichannel or multichannel retailing?  *“Moreover, while omnichannel and multichannel strategies are primarily focused on integration and management of distribution channels, the phygital customer experience (PH-CX) framework moves beyond this channel logic [4, 9]. With this context, physical and digital elements are intentionally combined to create a seamless continuum of value across online and offline settings. This definition follows Batat’s conceptual framework, which positions phygital as a consumer-centered ecosystem rather than a simple channel strategy. Phygital is more than the mere connection of points of sale; it represents a holistic ecosystem that makes the customer journey seamless across physical and digital domains. Contrastingly, omnichannel emphasizes transactional continuity, phygital prioritizes a sustained continuous stream of values such as financial, symbolic, social, and affective which are delivered via hybrid experiences that fuse sensory design, technology, and experiential features [10, 11]. Aligning with this, phygital should be operationalized as an experience construct, and not as a channel-management label.”* |
| 3. The study uses TAM3 and Service-Dominant Logic as theoretical foundations. While both are relevant, the manuscript could explain more explicitly how these frameworks are integrated to support the proposed model and hypotheses. | We appreciate this comment and have made the suggested improvement. In response, lines 233 - 239 have been revised to provide an explanation to how these adopted frameworks are relevant as per your comment,  *“Moreover, TAM3 demonstrates a close view to understand individual opinions such as PU, PEOU and DL which drive towards CE along with PE. In contrast, SDL enhances this perspective through embedding the adoption drivers within a holistic service ecosystem where customers serve as operant resources, value co production via CI and engagement. TAM3 constructs are viewed not solely as drivers of technology acceptance but also a mechanism of co production of value, reflecting SDL’s emphasis on resource integration.”*  Hence to support the frameworks, the proposed model and hypothesis are given below,  (Lines 259 – 266) H_1_ **–** “*Furthermore, TAM3 highlights that PU and PEOU are core to determine CE through technology. For instance, when users experience an innovative service setting, their level of involvement is identified by the extent to which they understand interaction as relevant, seamless, and compatible with their expectations [46]. TAM3 delivers justification at the operational level, where CE arises as a response behavior towards positive intellectual reviews of the system. By implementing TAM3, the relationship is reinforced through the reasoning that positive user perceptions of technology can directly translate to greater extent of CE.”*  (Lines 286 – 292) H_2_ ***–*** *“In TAM3, self-efficacy reduces perceived difficulty and increases the PEOU, thereby elevates the overall effectiveness of PE, where customers tend to purchase repeatedly from the same brand [50]. Hence, this improved self‑efficacy paves the way for more favorable cognitive evaluations of usefulness and ease of use, which directly promotes engagement behaviors [51]. This way, TAM3 provides theoretical justification for H2 by exhibiting that DL acts as a cognitive enabler, mediating the effect of system experience on CE*.”  (Lines 320 – 323) H_3 –_ *“CE also represents a customer’s ability and willingness to invest time, effort and knowledge in interactions and SDL emphasizes that such resource combination is the foundation of innovation in service ecosystems [58]. Thus, SDL shows that CE is a vital antecedent of CI, as it enables the active participation and knowledge sharing necessary for COC [59]*.”  (Lines 343 – 348) H_4_ ***–*** *“Within SDL, knowledge and creativity turn into a service ecosystem, in which their participation translates CI to COC, by enhancing organizational values and customer loyalty [66]. Moreover, when customers innovate, they extend the scope of resource integration by introducing novel approaches that reshape interactions and enhance collective outcomes [67]. Where, SDL identifies that CI is not an isolated activity but a critical antecedent of COC within service ecosystems.*”  (Lines 360 – 365) H_5 –_ “where customers see the outcomes which they helped shape. In the meantime, SDL directly tries COC to value perception, *and then SDL declares that value is not deep-seated in products or services but is co-created through interactions among active participants in an integrated service network [36]. Moreover, SDL highlights that PV is a relational construct emerging from the COC process rather than being delivered one-sided by firms [72].*”  (Lines 385 – 393) H_6_ – “*TAM3 emphasizes that PU and PEOU are crucial to shape user evaluations of technology; it also explains that CE reflects the behavioral manifestation of these positive cognitive evaluations, as individuals who perceive the respective system as useful and intuitive, are more likely to invest effort and attention in their engagement [79]. Such engagement enhances the value perception because customers experience outcomes perfectly align with their expectations and deliver efficiency and effectiveness. This way, TAM3 explains that PV emerges as a direct consequence of engagement behaviors rooted in favorable cognitive assessments of usefulness and ease of use.*”  (Lines 409 – 415) H_7 -_ “Good customer service, real-time quick support, and especially a user-friendly system reduces the barriers to shopping and thus build brand trust and SDL emphasizes value experiential as a driver of satisfaction *and conceptualizes that value is co-created through interactions among active participants rather than delivered unilaterally by firms. CS thus emerges as the affective consequence of PV, reinforcing SDL’s proposition that value creation is inherently tied to customer participation and COC processes* [2].”  (Lines 424 – 429) H_8_ – “SDL also explains that satisfaction is an antecedent of loyalty for exchange in service, and. *It also serves as an empirical foundation for long-term relationships, where satisfied customers identify that their anticipated outcomes have been met through operant resource integration, which consolidates their intention to continue the relationship. Therefore, SDL shows that CL is a logical progression of CS*.”  (Lines 449 – 454) H_9_ – “*TAM3 identifies that DL strengthens PU and PEOU, by facilitating customers to engage assertively with phygital systems. This cognitive structure triggers engagement that transitions to SDL, where engaged customers facilitate CI and COC. When customers gain greater value, it strengthens CS as a positive result [72]. Thus, TAM3 supports the initial integration point of DL and CE, while SDL explains the connection chain leading to CL*.”  (Lines 468 – 471) H_10_ – “*The relationship justifies TAM3 at the entry point of DL, where DL fortifies self-efficacy and weakens perceived complexity by enabling customers to confidently interact with PE. Additionally, SDL labels proactive involvement as resource incorporation which enhances PV driving it to CS in response to co-created outcomes and sustaining CL [2]*.”  (Lines 484 – 488) H_11_ – “*SDL supports this serial mediation,* [94] *explains that CE functions as a catalyst for integration of resources where it encourages CI within service delivery. CI gradually transforms into COC, in which customers work together with firms to tailor experiences to meet user needs* [56]*. These co-produced outcomes tend to increase PV, and higher the PV it increases CS where satisfied customers are more likely to be loyal* [95].  (Lines 502 – 505) H_12_ – “*SDL assists this serial mediation because CE elevates PV, since customers examine the end results more optimistically., because Hence, they identify their role is shaping the outcomes, in which thus increases CS leading towards a long-term CL [99]*.” |
| 4. The hypotheses are logically structured, but some sections rely heavily on citations rather than analytical reasoning. Strengthening the theoretical arguments linking the constructs would improve the rigor of hypothesis development. | We sincerely appreciate this constructive feedback.   A. We have revised selected sections of hypothesis development by reducing over-reliance on direct citations and improving analytical reasoning.   (Line 300 – 304) **“***Active CE exceeds beyond transaction-based involvement to sharing ideas, feedback, self-initiated actions in a manner that benefit both the customers and organizations. In SDL, such integration of resources can enable the organization to co-create value and drive towards CI, where active participation does not only benefit the organizations, but it enriches the overall output within the community [42].”*  (Line 339 – 342) **“***CI indicates engaged participation where customers share insights, form ideas, and collaborate with organizations. This user driven behavior not just encourages new solutions, but it also enhances purchase intentions, where customers develop stronger connection to the end results in which they helped shape [22].”*  (Line 283 – 288) **“**Moreover, [40], have identified that customers with high digital technology knowledge will increase the possibility of them buying from the brand repeatedly, *and it is because customers with a high DL tend to form a stronger self-efficacy and consider digital interactions as less complex. In TAM3, self-efficacy reduces perceived difficulty and increases the PEOU, thereby elevates the overall effectiveness of PE, where customers tend to purchase repeatedly from the same brand [50].*”   B. We have strengthened the theoretical arguments linking the constructs which would help to improve the rigor of hypothesis development.   (Lines 259 – 266) H_1_ **–** “Furthermore, TAM3 highlights that PU and PEOU are core to determine CE through technology. For instance, when users experience an innovative service setting, their level of involvement is identified by the extent to which they understand interaction as relevant, seamless, and compatible with their expectations [46]. TAM3 delivers justification at the operational level, where CE arises as a response behavior towards positive intellectual reviews of the system. By implementing TAM3, the relationship is reinforced through the reasoning that positive user perceptions of technology can directly translate to greater extent of CE*.”*  (Lines 286 – 292) H_2_ ***–*** *“In TAM3, self-efficacy reduces perceived difficulty and increases the PEOU, thereby elevates the overall effectiveness of PE, where customers tend to purchase repeatedly from the same brand [50]. Hence, this improved self‑efficacy paves the way for more favorable cognitive evaluations of usefulness and ease of use, which directly promotes engagement behaviors [51]. This way, TAM3 provides theoretical justification for H2 by exhibiting that DL acts as a cognitive enabler, mediating the effect of system experience on CE*.”  (Lines 320 – 323) H_3 –_ *“CE also represents a customer’s ability and willingness to invest time, effort and knowledge in interactions and SDL emphasizes that such resource combination is the foundation of innovation in service ecosystems [58]. Thus, SDL shows that CE is a vital antecedent of CI, as it enables the active participation and knowledge sharing necessary for COC [59]*.”  (Lines 343 – 348) H_4_ ***–*** *“Within SDL, knowledge and creativity turn into a service ecosystem, in which their participation translates CI to COC, by enhancing organizational values and customer loyalty [66]. Moreover, when customers innovate, they extend the scope of resource integration by introducing novel approaches that reshape interactions and enhance collective outcomes [67]. Where, SDL identifies that CI is not an isolated activity but a critical antecedent of COC within service ecosystems.*”  (Lines 360 – 365) H_5 –_ “where customers see the outcomes which they helped shape. In the meantime, SDL directly tries COC to value perception, *and then SDL declares that value is not deep-seated in products or services but is co-created through interactions among active participants in an integrated service network [36]. Moreover, SDL highlights that PV is a relational construct emerging from the COC process rather than being delivered one-sided by firms [72].*”  (Lines 385 – 393) H_6_ – “*TAM3 emphasizes that PU and PEOU are crucial to shape user evaluations of technology; it also explains that CE reflects the behavioral manifestation of these positive cognitive evaluations, as individuals who perceive the respective system as useful and intuitive, are more likely to invest effort and attention in their engagement [79]. Such engagement enhances the value perception because customers experience outcomes perfectly align with their expectations and deliver efficiency and effectiveness. This way, TAM3 explains that PV emerges as a direct consequence of engagement behaviors rooted in favorable cognitive assessments of usefulness and ease of use.*”  (Lines 409 – 415) H_7 -_ “Good customer service, real-time quick support, and especially a user-friendly system reduces the barriers to shopping and thus build brand trust and SDL emphasizes value experiential as a driver of satisfaction *and conceptualizes that value is co-created through interactions among active participants rather than delivered unilaterally by firms. CS thus emerges as the affective consequence of PV, reinforcing SDL’s proposition that value creation is inherently tied to customer participation and COC processes* [2].”  (Lines 424 – 429) H_8_ – “SDL also explains that satisfaction is an antecedent of loyalty for exchange in service, and. *It also serves as an empirical foundation for long-term relationships, where satisfied customers identify that their anticipated outcomes have been met through operant resource integration, which consolidates their intention to continue the relationship. Therefore, SDL shows that CL is a logical progression of CS*.”  (Lines 449 – 454) H_9_ – “*TAM3 identifies that DL strengthens PU and PEOU, by facilitating customers to engage assertively with phygital systems. This cognitive structure triggers engagement that transitions to SDL, where engaged customers facilitate CI and COC. When customers gain greater value, it strengthens CS as a positive result [72]. Thus, TAM3 supports the initial integration point of DL and CE, while SDL explains the connection chain leading to CL*.”  (Lines 468 – 471) H_10_ – “*The relationship justifies TAM3 at the entry point of DL, where DL fortifies self-efficacy and weakens perceived complexity by enabling customers to confidently interact with PE. Additionally, SDL labels proactive involvement as resource incorporation which enhances PV driving it to CS in response to co-created outcomes and sustaining CL [2]*.”  (Lines 484 – 488) H_11_ – “*SDL supports this serial mediation,* [94] *explains that CE functions as a catalyst for integration of resources where it encourages CI within service delivery. CI gradually transforms into COC, in which customers work together with firms to tailor experiences to meet user needs* [56]*. These co-produced outcomes tend to increase PV, and higher the PV it increases CS where satisfied customers are more likely to be loyal* [95].  (Lines 502 – 505) H_12_ – “*SDL assists this serial mediation because CE elevates PV, since customers examine the end results more optimistically., because Hence, they identify their role is shaping the outcomes, in which thus increases CS leading towards a long-term CL [99]*.” |
| 5. It is suggested to add article entitled “Kondybayeva et al. A New Concept of Transforming Service: Impact of Generative Voice Chatbots on Customer Satisfaction and Banking Industry Productivity” to the literature review. | Thank you for your valuable comment. We appreciate your feedback and we have added the article to our study. In line 434 to 438 as follows,  **“**Moreover*, in phygital settings the direct path is crucial; as explained previously if customers perceive that omnichannel experiences sustain performance aligned with expectations, they tend to maintain a lasting relationship. Through empirical findings in digital banking proves that CS, along with generative AI chatbots explicitly boosts loyalty outcomes by consolidating confidence and sustained adoption* [89].” |
| 6. The use of purposive sampling to collect responses from Gen Z customers is understandable, but it may limit generalizability. The authors should discuss potential sampling bias and justify why this approach was most appropriate for the study | We appreciate this valuable comment. The purposive sampling was intentionally used due to this study specially targets Gen Z consumers who are highly engaged with phygital retail environments. The investigation aims to examine customer engagement and innovation behaviors within a digitally active segment, so purposive sampling is appropriate for selecting respondents who have relevant experience and familiarity with such contexts. In lines, 527-533 added the changes of the manuscript as follow,  “The purposive sampling method, which was used by [15] helped to capture the respondents who experienced phygital aspects in their buying process, and the sample consisted of 480 respondents. *This study specially targeted respondents who engaged with phygital experience related activities during their shopping journey. Through the study, authors aims to examine customer engagement and innovation behaviors within the specific content. The selected, this sampling approach enables the selection of respondents with relevant experience, thereby improving the validity of responses.*”  However, we acknowledge that purposive sampling may introduce sampling bias and limit the generalizability of the findings to the broader population. The limitation section has now been explicitly discussed in the manuscript in lines 903-905. Future studies are encouraged to employ probability sampling techniques or include a more diverse demographic to enhance generalizability.  *“Finally, as the purposive sample technique chosen in this study may generate sample bias and limit generalizability, probability sampling techniques can be used for future investigations in broader populations.”* |
| 7. The manuscript mentions that questionnaire items were adapted from prior studies. It would be useful if the authors explained how these items were modified to fit the phygital retail context and whether a pilot test was conducted. | Thank you for this valuable suggestion. We have now clarified how the questionnaire items were adapted from prior studies to suit the phygital retail context, In 551-564 lines are included the process of modifying the questionnaire items to be fitted to the study context.  “To investigate the above-mentioned casual relationships from the correct target population, the questionnaire relied on the three (3) main layers. The first layer included the demographic information of the participants of the survey, the second layer had four (04) main questions for filtering known as purposive questions. *“1. Have you used services/products that combine physical and digital experiences?”, “2. Have you ever given feedback or suggestions about a product or service you purchased?” “3. Have you purchased products or services from the brand/shop/retailer in the last 6 months?”, “4. Have you recommended a product or store to someone else based on your experience?”*. The final layer consists of dimension validation questions with *a 5-point Likert scale. These second and final layers were modified according to the contextual situation and improved practicality from the customer’s perspective. Before finalizing the survey questions, a pilot test with 70 responses was conducted to confirm the questions and their practical usability in customer perception. Then, inconsistencies were identified and questions were re-modified to obtain a valid and reliable output.*” |
| 8. Data were collected through an online questionnaire distributed via QR codes. While practical, this method might exclude less digitally engaged consumers; the authors should discuss how this may influence the sample composition. | Thank you for the insightful comment. The questionnaire was distributed via QR code, this method was chosen for its practicality and alignment with the study context, particularly as the research focuses on digitally engaged Gen Z consumers within the phygital retail environments. In 537-546 lines are discussing further how this method influence the chosen sample composition.  “The survey questionnaire fulfilled requirements and qualifications to do an investigation, the online method was chosen for cost-effectiveness, ease of distribution, and suitability for urban areas [87]. For that QR code scanning facilities was used to distribute the survey link, allowing respondents to easily access and complete the questionnaire using their mobile devices. *Despite this, the use of QR code-based online data collection may have influenced the sample composition by identifying the respondents who are more digitally literate and more comfortable with mobile-based interactions. Simultaneously, the sample may have overrepresented digitally connected Gen Z consumers, while underrepresenting individuals with low levels of digital access or familiarity (i.e. digital divide).*” |
| 9. It is also suggested to add articles entitled “Angkhasakulkiat et al. Digital Literacy for Business Performance: A Study of Entrepreneurs” and “T. K. Q. Nguyen. E-Banking Service Quality and Customer Satisfaction with Moderator Factor” to the literature review. | We appreciate this comment and have incorporated the suggested improvement. In line 274 - 279 as follows,  **“***Furthermore, DL is a strategic ability that enables customers to respond and utilize digital tools effectively. The mediating role guarantees that PE transforms into deeper level of engagement, where customers who are tech – savvy can better recognize opportunities in dynamic environments, Furthermore it also confirms that DL functions as highly influential mediating construct in between digital capabilities and business outcomes which highlights its importance in contributing to engagement* [48].”  In lines 400 - 405 as follows,  **“***PV is also enhanced by service quality constructs such as responsiveness, confidentiality, and reliability, and not solely by co-creation. The evidence is supported from an e-banking study, it showcasing efficiency, reliability, responsiveness and confidentiality; it all indicates positive and significant effect on e-banking services, which in turn inferred had a strong positive impact on CS [83]*.” |
| 10. The measurement model shows some factor loadings below 0.70 that were retained. The authors briefly justify this decision, but additional explanation about the theoretical relevance of these items would strengthen the validity argument. | Thank you for this valuable comment. We acknowledge that several indicator loadings were below the recommended threshold of 0.70. While higher loadings are generally preferred, indicators with loading between 0.60 and 0.70 were retained as they meet acceptable thresholds and do not adversely affect composite reliability or average variance extracted (AVE). In 621-628 lines addressed the further justification on the manuscript as follows.  “Most of the loadings were above 0.70, supporting indicator loadings. Except for a few items, such as CE1 (0.635), CE5 (0.667), and CI1 (0.659) exhibited relatively lower loadings. Aligning with the argument of [106, 107], those items were retained due to their acceptable acceptance level and satisfactory convergent validity (AVE > 0.50). *Furthermore, these items were theoretically important in capturing the entire domain of the constructs, especially for multidimensional variables like CE and CI. Removing these items would compromise the content validity of the measurement model, and* using Cronbach’s alpha (α), composite reliability rho_a, and rho_c internal consistency reliability was assessed.” |
| 11. The use of PLS-SEM is appropriate for complex models with multiple mediators. However, the manuscript could provide a stronger justification for choosing PLS-SEM instead of covariance-based SEM. | Thank you for the valuable comment, we would like to address that choosing PLS-SEM model instead of any other models due to its own characteristics, and it matched with our study objectives. Apart from that the non-normal data distribution nature explicit that PLS-SEM is more accurate than CB-SEM. In 582-586 lines we addressed the justification for choosing PLS-SEM. The updated lines are as follows.  “The Structural Equation Modeling (SEM) with Partial Least Squares (PLS) was used to analyses via SmartPLS 4.1.1.4 software, a suitable technique for explanatory models involving multiple constructs, and dimensions [121]. *Apart from other analytical techniques, PLS-SEM model aligns with research objectives, fits data characteristics and suits model complexity.*” |
| 12. The model includes multiple serial mediation paths involving several constructs. While interesting, the manuscript should clarify whether all mediators are theoretically necessary or whether the model risks becoming overly complex. | We thank the reviewer for this valuable observation. The inclusion of multiple serial mediation hypotheses (H_9_–H_12_) were not intended to overcomplicate the model, but rather to systematically examine the theoretical necessity of each mediator.  In our study, each mediator was included based on prior theoretical and empirical evidence suggesting a sequential mechanism linking the independent and dependent variables.  To address the concern regarding model complexity, we have revised the manuscript to more clearly justify the inclusion and sequence of each mediator in the discussion section.  In lines 868-880 we addressed the justification for choosing multiple serial mediation.  “*The integration of the serial mediation hypothesis was proposed to identify the integrated mechanisms through which PE determines CL. Each hypothesis symbolizes a separate yet complementary pathway. The expanded sequence in H_9_ shows how DL promotes CE, which then encourages CI and COC, eventually leading to PV, CS and CL. H_10_ tests whether DL and CE are solely capable of driving value and satisfaction, therefore it clarifies the role of CI and COC, while H_11_ identifies customer influenced mechanisms of CE, CI, and COC, which show that CL can appear even without DL as an initial step. Finally, H_12_ portrays the simplest SDL pathway, where CE directly affects value perception and CS, leading to CL. Together, these serial mediation hypotheses enable the model to differentiate between holistic and simplified processes, which ensures that mediators are not chosen randomly but are experimentally evaluated for necessity. The results validate that shorter chains represent essential dynamics, but the extended path provides a comprehensive explanatory power, by justifying the serial mediation hypothesis in the study*.” |
| **2^nd^ Reviewers’ Comment** | **Response to the Comment** |
| 1. The topic is timely and potentially valuable, particularly because it examines phygital retailing, Gen Z, and an emerging-market context; however, the manuscript requires major revision before it can be considered further. My main concerns are that the paper contains important inconsistencies in reporting and transparency, including conflicting data-availability statements, unclear final analytic sample size because the manuscript reports 480 respondents while the measurement model appears to use 419 cases, and several apparent errors or inconsistencies in the SEM reporting, tables, and interpretations that need to be carefully checked and corrected. In addition, the methods section needs substantially more detail to support reproducibility, especially regarding recruitment, screening, filtering questions, exclusions, and handling of response quality, while the mediation results and hypothesis numbering should be reviewed for consistency across the theory, results, and discussion sections. The manuscript also needs thorough language editing and technical cleanup, including correction of unresolved cross-references, and the conclusions should be phrased more cautiously because the study is based on cross-sectional, self-reported data and therefore supports associations rather than strong causal claims. Overall, the study has potential, but substantial revision is needed in methodological clarity, reporting accuracy, and presentation quality before its contribution can be properly evaluated. | Thank you addressing the issue on this section. The study sample technique that employed was purposive sampling, in the survey there was a Section (B), it contained 4 filtering questions, the most and crucial important question was “01. Have you tried Phygital experiences that combine physical and digital experiences while shopping?”. After the respondents were filtered and taken to the analysis. Even though there are 480 respondents, after filtering the respondents reduced to 419, because their shopping experience and behaviors can explicit a deep understanding and suggestion to the consumer electronics retail sector. The respondent who gave yes to the main filtered question only taken to the data analysis and the filtered sample count were showed in the Table 2. Demographic information. For further understanding, lines 597 - 607 were added as follows,  “The study reveals a diverse demographic, with a majority of respondents were male 56.67% while females accounted for 43.33%, indicating fairly balanced gender representation. Young adults dominate with 66.67% aged 22-25. Most respondents have a bachelor’s degree or undergraduate , with 86.88% having advanced degrees. The highest occupation is students, with 26.88% employed and 6.88% self-employed. *Furthermore, 67.29% of respondents are having* less than LKR 50,000 *income*. Exploring that monthly (29.79%) and rare (35%) purchases were most common, with 87.29% of respondents engaging in phygital experience during their buying process. *Along with this, 419 out of 480 respondents were taken into the analysis due to the minimum requirement subject. This is because the study only considers valid data from respondents who have gained phygital experience during their shopping journey.*”  After thorough investigating, we added further information on how we choose questionnaire items, screening, filtering questions based on the contextual relevance & practicality, and included the filtering questions that allowed handling of response quality.  Then after proofreading the document, we reviewed the consistency of the hypothesis numbering and did the changes in mentioned sections. The correction process of language editing, technical cleanup, unsolved cross-reference was reviewed and corrected. Regarding the conclusion, we assume that the reviewer’s suggestion is relevant to the nature of the study, which was cross-sectional, so we remodified the conclusion in lines between 914-940 the corrected one is as follows.  *“The study contributes to the body of knowledge on hybrid retail ecosystems in an emerging market setting by providing empirical evidence that PE can be a strong driver of CL among Gen Z consumers in the consumer electronics retail sector of Sri Lanka. The PLS-SEM analysis inferred all the 12 hypothesized relationships. It means that PE has a positive and significant impact on CE, which in turn, activates the sequential value-creation chain through CI, COC, PV and CS and finally, leads to CL. One of the theoretical contributions of this paper is the empirical validation of DL as a significant mediator between PE and CE. This observation confirms that PE in isolation cannot achieve engagement unless the customers have sufficient digital skills to navigate technology-based retail space. Moreover, four serial mediation pathways established confirm that loyalty in phygital contexts is formed through a cumulative, multi-stage psychological process, but not through any specific, direct mechanism. In essence, these results provide pragmatic advice to retailers doing business in the emerging economies. Managers are not only to invest in the process of integrating AR/VR, IoT, and seamless digital/physical touchpoints, but also in building the DL of customers by providing in-store training, creating user-friendly interface design, and implementing technology onboarding programmers. PV and long-term loyalty can be further enhanced by fostering COC opportunities. This study adds to the extant literature of phygital by introducing new mediating variables in one integrated framework beyond previous studies on single mediating variables. It is also contextual to phygital adoption in a developing country like Sri Lanka (where political and economic instability are prevalent) and can be transferred to other emerging markets. However, the scope of the study is limited by the cross-sectional design, use of self-reported data, limited to one industry and a single demographic, as well as geographically to Sri Lanka. Future studies warrant the use of longitudinal designs and be extended to cross-cutting industries, age groups and even diverse cultural environments. The inclusion of such variables as consumer trust, emotional responses, and digital fatigue would further enrich theoretical frameworks of phygital loyalty formation.”*  We acknowledge that there are major revisions happened to improve methodological clarity, reporting accuracy, and presenting quality of the current manuscript.  Finally, we acknowledge that the data sets regarding this investigation such as survey questionnaire, QR code, response recorded sheet, and data sheet of the analysis are available on request for the journal authorities. |
| **3^rd^ Reviewer’s comment** | **Response to the Comment** |
| The paper lacks relevant statistical tests. The paper lacks amthematical support to the proposed method. A figure for the entire framework with details is missing in the paper. The paper lacks sufficient contribution for publication. | Thank you for the valuable review comment. The identified missing PLS predict test was added to the manuscript and it is on 696-703 lines as follow. The results of the PLS predict were combined with the table 8, R^2^ and adjusted R^2^ values mentioned table.  “*The PLSpredict procedure use to assess predictive validity, which measures the out of sample predictive performance of the model. The findings emphasize that all endogenous constructs show a positive Q2 prediction value by confirming the existence of predictive relevance. CE (0.634) and DL (0.638) both indicate strong predictive power with Q2 predict values exceeding 0.60, while CI, CL, CS, and PV show moderate predictive values. However, COC with a small predictive value (0.191), even though is negligible, demonstrates predictive capability.*”  After that, the Q^2^ predictive values were included into the table no:08, the R^2^ and adjusted R^2^ table.  “*Q2 Predictive*  *CE - 0.634*  *CI - 0.421*  *CL - 0.307*  *COC - 0.191*  *CS - 0.396*  *DL - 0.638*  *PV - 0.465* ”  Thank you for addressing the fault . According to the journal guidelines, the figures should be handover separately, not in the manuscript, so we didn’t include that into the manuscript, instead of that, it was separately attached as a supported document file and mentioned the figure number in the manuscript. We acknowledge that the paper was revised again and corrected all the possible mistakes that have been addressed by the reviewers. We addressed the lack of theoretical and methodological clarity of the manuscript and made sure to do a revision on the manuscript especially on method section, now we are believing that this manuscript suits for a publication. |
