# Supplementary material for: Unlocking Gen Z engagement in phygital retailing: Evidence from a multi-mediated customer experience model
Source: PLoS One. 2026 Aug 6;21(8):e0353490. doi: 10.1371/journal.pone.0353490 (PMC13446700; doi:10.1371/journal.pone.0353490)
Supplement: Peer review history

# Peer review: Decision Letter 0

**DOI:** 10.1371/journal.pone.0353490.r001

**Authors:** Rajesh Kumar

21 Apr 2026

PONE-D-26-05131

The complete manuscript Unlocking Gen Z Engagement in Phygital Retailing: Evidence from a Multi-Mediated Customer Experience Model

PLOS One

Dear Dr. Wisenthige,

Thank you for submitting your manuscript to PLOS ONE. After careful consideration, we feel that it has merit but does not fully meet PLOS ONE’s publication criteria as it currently stands. Therefore, we invite you to submit a revised version of the manuscript that addresses the points raised during the review process.

Please submit your revised manuscript by Jun 05 2026 11:59PM. If you will need more time than this to complete your revisions, please reply to this message or contact the journal office at plosone@plos.org. When you're ready to submit your revision, log on to https://www.editorialmanager.com/pone/ and select the 'Submissions Needing Revision' folder to locate your manuscript file.

Please include the following items when submitting your revised manuscript:

- A letter that responds to each point raised by the academic editor and reviewer(s). You should upload this letter as a separate file labeled 'Response to Reviewers'.
- A marked-up copy of your manuscript that highlights changes made to the original version. You should upload this as a separate file labeled 'Revised Manuscript with Track Changes'.
- An unmarked version of your revised paper without tracked changes. You should upload this as a separate file labeled 'Manuscript'.

If you would like to make changes to your financial disclosure, please include your updated statement in your cover letter. Guidelines for resubmitting your figure files are available below the reviewer comments at the end of this letter.

If applicable, we recommend that you deposit your laboratory protocols in protocols.io to enhance the reproducibility of your results. Protocols.io assigns your protocol its own identifier (DOI) so that it can be cited independently in the future. For instructions see: https://journals.plos.org/plosone/s/submission-guidelines#loc-laboratory-protocols. Additionally, PLOS ONE offers an option for publishing peer-reviewed Lab Protocol articles, which describe protocols hosted on protocols.io. Read more information on sharing protocols at https://plos.org/protocols?utm_medium=editorial-email&utm_source=authorletters&utm_campaign=protocols.

As the corresponding author, your ORCID iD is verified in the submission system and will appear in the published article. PLOS supports the use of ORCID, and we encourage all coauthors to register for an ORCID iD and use it as well. Please encourage your coauthors to verify their ORCID iD within the submission system before final acceptance, as unverified ORCID iDs will not appear in the published article. *Only*  the individual author can complete the verification step; PLOS staff *cannot*  verify ORCID iDs on behalf of authors.

We look forward to receiving your revised manuscript.

Kind regards,

Dr. Rajesh Kumar

Academic Editor

PLOS One

Journal Requirements:

When submitting your revision, we need you to address these additional requirements.

1. Please ensure that your manuscript meets PLOS ONE's style requirements, including those for file naming. The PLOS ONE style templates can be found at

https://journals.plos.org/plosone/s/file?id=wjVg/PLOSOne_formatting_sample_main_body.pdf and

https://journals.plos.org/plosone/s/file?id=ba62/PLOSOne_formatting_sample_title_authors_affiliations.pdf

2. Thank you for submitting the above manuscript to PLOS ONE. During our internal evaluation of the manuscript, we found significant text overlap between your submission and previous work in the [introduction, conclusion, etc.].

We would like to make you aware that copying extracts from previous publications, especially outside the methods section, word-for-word is unacceptable. In addition, the reproduction of text from published reports has implications for the copyright that may apply to the publications.

Please revise the manuscript to rephrase the duplicated text, cite your sources, and provide details as to how the current manuscript advances on previous work. Please note that further consideration is dependent on the submission of a manuscript that addresses these concerns about the overlap in text with published work.

[If the overlap is with the authors’ own works: Moreover, upon submission, authors must confirm that the manuscript, or any related manuscript, is not currently under consideration or accepted elsewhere. If related work has been submitted to PLOS ONE or elsewhere, authors must include a copy with the submitted article. Reviewers will be asked to comment on the overlap between related submissions (http://journals.plos.org/plosone/s/submission-guidelines#loc-related-manuscripts).]

We will carefully review your manuscript upon resubmission and further consideration of the manuscript is dependent on the text overlap being addressed in full. Please ensure that your revision is thorough as failure to address the concerns to our satisfaction may result in your submission not being considered further

3. Your ethics statement should only appear in the Methods section of your manuscript. If your ethics statement is written in any section besides the Methods, please delete it from any other section.

4. In this instance it seems there may be acceptable restrictions in place that prevent the public sharing of your minimal data. However, in line with our goal of ensuring long-term data availability to all interested researchers, PLOS’ Data Policy states that authors cannot be the sole named individuals responsible for ensuring data access (http://journals.plos.org/plosone/s/data-availability#loc-acceptable-data-sharing-methods).

Data requests to a non-author institutional point of contact, such as a data access or ethics committee, helps guarantee long term stability and availability of data. Providing interested researchers with a durable point of contact ensures data will be accessible even if an author changes email addresses, institutions, or becomes unavailable to answer requests.

Before we proceed with your manuscript, please also provide non-author contact information (phone/email/hyperlink) for a data access committee, ethics committee, or other institutional body to which data requests may be sent. If no institutional body is available to respond to requests for your minimal data, please consider if there any institutional representatives who did not collaborate in the study, and are not listed as authors on the manuscript, who would be able to hold the data and respond to external requests for data access? If so, please provide their contact information (i.e., email address). Please also provide details on how you will ensure persistent or long-term data storage and availability.

5. If the reviewer comments include a recommendation to cite specific previously published works, please review and evaluate these publications to determine whether they are relevant and should be cited. There is no requirement to cite these works unless the editor has indicated otherwise.

[Note: HTML markup is below. Please do not edit.]

Reviewers' comments:

Reviewer's Responses to Questions

**Comments to the Author**

1. Is the manuscript technically sound, and do the data support the conclusions?

The manuscript must describe a technically sound piece of scientific research with data that supports the conclusions. Experiments must have been conducted rigorously, with appropriate controls, replication, and sample sizes. The conclusions must be drawn appropriately based on the data presented.

Reviewer #1: Partly

Reviewer #2: Yes

Reviewer #3: No

**********

2. Has the statistical analysis been performed appropriately and rigorously?

Reviewer #1: Yes

Reviewer #2: Yes

Reviewer #3: No

**********

3. Have the authors made all data underlying the findings in their manuscript fully available?

The PLOS Data policy requires authors to make all data underlying the findings described in their manuscript fully available without restriction, with rare exception (please refer to the Data Availability Statement in the manuscript PDF file). The data should be provided as part of the manuscript or its supporting information, or deposited to a public repository. For example, in addition to summary statistics, the data points behind means, medians and variance measures should be available. If there are restrictions on publicly sharing data—e.g. participant privacy or use of data from a third party—those must be specified.

Reviewer #1: Yes

Reviewer #2: Yes

Reviewer #3: Yes

**********

4. Is the manuscript presented in an intelligible fashion and written in standard English?

PLOS ONE does not copyedit accepted manuscripts, so the language in submitted articles must be clear, correct, and unambiguous. Any typographical or grammatical errors should be corrected at revision, so please note any specific errors here.

Reviewer #1: Yes

Reviewer #2: Yes

Reviewer #3: Yes

**********

5. Review Comments to the Author

Please use the space provided to explain your answers to the questions above. You may also include additional comments for the author, including concerns about dual publication, research ethics, or publication ethics. (Please upload your review as an attachment if it exceeds 20,000 characters)

Reviewer #1: 1.The study focuses on Sri Lanka’s consumer electronics retail sector, which is valuable for emerging market insights. However, the manuscript could better justify why this context is particularly suitable for studying phygital experiences and Gen Z engagement.

2.The concept of phygital experience is introduced well, but the manuscript could benefit from a clearer operational definition. How do the authors distinguish phygital experience from omnichannel or multichannel retailing?

3.The study uses TAM3 and Service-Dominant Logic as theoretical foundations. While both are relevant, the manuscript could explain more explicitly how these frameworks are integrated to support the proposed model and hypotheses.

4.The hypotheses are logically structured, but some sections rely heavily on citations rather than analytical reasoning. Strengthening the theoretical arguments linking the constructs would improve the rigor of hypothesis development.

5.It is suggested to add article entitled “Kondybayeva et al. A New Concept of Transforming Service: Impact of Generative Voice Chatbots on Customer Satisfaction and Banking Industry Productivity” to the literature review.

6.The use of purposive sampling to collect responses from Gen Z customers is understandable, but it may limit generalizability. The authors should discuss potential sampling bias and justify why this approach was most appropriate for the study.

7.The manuscript mentions that questionnaire items were adapted from prior studies. It would be useful if the authors explained how these items were modified to fit the phygital retail context and whether a pilot test was conducted.

8.Data were collected through an online questionnaire distributed via QR codes. While practical, this method might exclude less digitally engaged consumers; the authors should discuss how this may influence the sample composition.

9.It is also suggested to add articles entitled “Angkhasakulkiat et al. Digital Literacy for Business Performance: A Study of Entrepreneurs” and “T. K. Q. Nguyen. E-Banking Service Quality and Customer Satisfaction with Moderator Factor” to the literature review.

10.The measurement model shows some factor loadings below 0.70 that were retained. The authors briefly justify this decision, but additional explanation about the theoretical relevance of these items would strengthen the validity argument.

11.The use of PLS-SEM is appropriate for complex models with multiple mediators. However, the manuscript could provide a stronger justification for choosing PLS-SEM instead of covariance-based SEM.

12.The model includes multiple serial mediation paths involving several constructs. While interesting, the manuscript should clarify whether all mediators are theoretically necessary or whether the model risks becoming overly complex.

Reviewer #2: The topic is timely and potentially valuable, particularly because it examines phygital retailing, Gen Z, and an emerging-market context; however, the manuscript requires major revision before it can be considered further. My main concerns are that the paper contains important inconsistencies in reporting and transparency, including conflicting data-availability statements, unclear final analytic sample size because the manuscript reports 480 respondents while the measurement model appears to use 419 cases, and several apparent errors or inconsistencies in the SEM reporting, tables, and interpretations that need to be carefully checked and corrected. In addition, the methods section needs substantially more detail to support reproducibility, especially regarding recruitment, screening, filtering questions, exclusions, and handling of response quality, while the mediation results and hypothesis numbering should be reviewed for consistency across the theory, results, and discussion sections. The manuscript also needs thorough language editing and technical cleanup, including correction of unresolved cross-references, and the conclusions should be phrased more cautiously because the study is based on cross-sectional, self-reported data and therefore supports associations rather than strong causal claims. Overall, the study has potential, but substantial revision is needed in methodological clarity, reporting accuracy, and presentation quality before its contribution can be properly evaluated.

Reviewer #3: The paper lacks relevant statistical tests. The paper lacks amthematical support to the proposed method. A figure for the entire farmework with details is missing in the paper. The paper lacks sufficient contribution for publication.

**********

6. PLOS authors have the option to publish the peer review history of their article (what does this mean?). If published, this will include your full peer review and any attached files.

If you choose “no”, your identity will remain anonymous but your review may still be made public.

**Do you want your identity to be public for this peer review?**  For information about this choice, including consent withdrawal, please see our Privacy Policy.

Reviewer #1: No

Reviewer #2: **Yes:**  Assoc.Prof.Dr.Nath Amornpinyo

Reviewer #3: No

**********

[NOTE: If reviewer comments were submitted as an attachment file, they will be attached to this email and accessible via the submission site. Please log into your account, locate the manuscript record, and check for the action link "View Attachments". If this link does not appear, there are no attachment files.]

To ensure your figures meet our technical requirements, please review our figure guidelines: https://journals.plos.org/plosone/s/figures

You may also use PLOS’s free figure tool, NAAS, to help you prepare publication quality figures: https://journals.plos.org/plosone/s/figures#loc-tools-for-figure-preparation.

NAAS will assess whether your figures meet our technical requirements by comparing each figure against our figure specifications.

# Author comment: Author response to Decision Letter 1

**DOI:** 10.1371/journal.pone.0353490.r002

6 Jun 2026

Please find the response to the reviewers' document provided.

Attachment Submitted filename: Author Response.docx

# Peer review: Decision Letter 1

**DOI:** 10.1371/journal.pone.0353490.r003

**Authors:** Rajesh Kumar

24 Jun 2026

The complete manuscript Unlocking Gen Z Engagement in Phygital Retailing: Evidence from a Multi-Mediated Customer Experience Model

PONE-D-26-05131R1

Dear Dr. Wisenthige,

We’re pleased to inform you that your manuscript has been judged scientifically suitable for publication and will be formally accepted for publication once it meets all outstanding technical requirements.

Within one week, you’ll receive an e-mail detailing the required amendments. When these have been addressed, you’ll receive a formal acceptance letter and your manuscript will be scheduled for publication.

An invoice will be generated when your article is formally accepted. Please note, if your institution has a publishing partnership with PLOS and your article meets the relevant criteria, all or part of your publication costs will be covered. Please make sure your user information is up-to-date by logging into Editorial Manager at Editorial Manager® and clicking the ‘Update My Information' link at the top of the page. For questions related to billing, please contact billing support.

If your institution or institutions have a press office, please notify them about your upcoming paper to help maximize its impact. If they’ll be preparing press materials, please inform our press team as soon as possible -- no later than 48 hours after receiving the formal acceptance. Your manuscript will remain under strict press embargo until 2 pm Eastern Time on the date of publication. For more information, please contact onepress@plos.org.

Kind regards,

Dr. Rajesh Kumar

Academic Editor

PLOS One

Additional Editor Comments (optional):

Reviewers' comments:

Reviewer's Responses to Questions

**Comments to the Author**

1. If the authors have adequately addressed your comments raised in a previous round of review and you feel that this manuscript is now acceptable for publication, you may indicate that here to bypass the “Comments to the Author” section, enter your conflict of interest statement in the “Confidential to Editor” section, and submit your "Accept" recommendation.

Reviewer #1: All comments have been addressed

Reviewer #2: All comments have been addressed

Reviewer #3: All comments have been addressed

**********

2. Is the manuscript technically sound, and do the data support the conclusions?

The manuscript must describe a technically sound piece of scientific research with data that supports the conclusions. Experiments must have been conducted rigorously, with appropriate controls, replication, and sample sizes. The conclusions must be drawn appropriately based on the data presented.

Reviewer #1: Yes

Reviewer #2: Yes

Reviewer #3: Yes

**********

3. Has the statistical analysis been performed appropriately and rigorously?

Reviewer #1: Yes

Reviewer #2: Yes

Reviewer #3: Yes

**********

4. Have the authors made all data underlying the findings in their manuscript fully available?

The PLOS Data policy requires authors to make all data underlying the findings described in their manuscript fully available without restriction, with rare exception (please refer to the Data Availability Statement in the manuscript PDF file). The data should be provided as part of the manuscript or its supporting information, or deposited to a public repository. For example, in addition to summary statistics, the data points behind means, medians and variance measures should be available. If there are restrictions on publicly sharing data—e.g. participant privacy or use of data from a third party—those must be specified.

Reviewer #1: Yes

Reviewer #2: Yes

Reviewer #3: Yes

**********

5. Is the manuscript presented in an intelligible fashion and written in standard English?

PLOS ONE does not copyedit accepted manuscripts, so the language in submitted articles must be clear, correct, and unambiguous. Any typographical or grammatical errors should be corrected at revision, so please note any specific errors here.

Reviewer #1: Yes

Reviewer #2: Yes

Reviewer #3: Yes

**********

6. Review Comments to the Author

Please use the space provided to explain your answers to the questions above. You may also include additional comments for the author, including concerns about dual publication, research ethics, or publication ethics. (Please upload your review as an attachment if it exceeds 20,000 characters)

Reviewer #1: The Revisions are satisfactory in my opinion, and I would certainly recommend the Editors to Publish the Paper in their esteemed Journal.

Reviewer #2: The revised manuscript has improved substantially and now presents a clearer and more coherent contribution to the literature on phygital retailing and Gen Z customer engagement. The topic is timely and relevant, particularly given the growing importance of integrating physical and digital retail touchpoints in emerging market contexts.

The authors have strengthened the conceptual framing and provided a more structured explanation of the relationships among phygital experience, digital literacy, customer engagement, customer innovation, co-creation, perceived value, satisfaction, and loyalty. The use of PLS-SEM is appropriate for examining the proposed multi-mediated model, and the sample size appears adequate for the empirical analysis. The findings are clearly presented and offer useful theoretical and managerial implications for retailers seeking to enhance customer loyalty through hybrid retail experiences.

Overall, the manuscript is well revised, and the authors appear to have addressed the previous comments constructively. I have no further major concerns. The manuscript is suitable for publication after any final editorial or formatting checks required by the journal.

Reviewer #3: The paper is good for publication as it has adequate contribution for publication. The revised version addresses the issues mentioned.

**********

7. PLOS authors have the option to publish the peer review history of their article (what does this mean?). If published, this will include your full peer review and any attached files.

If you choose “no”, your identity will remain anonymous but your review may still be made public.

**Do you want your identity to be public for this peer review?**  For information about this choice, including consent withdrawal, please see our Privacy Policy.

Reviewer #1: No

Reviewer #2: **Yes:**  Assoc.Prof.Dr.Nath Amornpinyo

Reviewer #3: No

**********

# Editor report: Acceptance letter

**DOI:** 10.1371/journal.pone.0353490.r004

**Authors:** Rajesh Kumar

PONE-D-26-05131R1

PLOS One

Dear Dr. Wisenthige,

I'm pleased to inform you that your manuscript has been deemed suitable for publication in PLOS One. Congratulations! Your manuscript is now being handed over to our production team.

At this stage, our production department will prepare your paper for publication. This includes ensuring the following:

* All references, tables, and figures are properly cited

* All relevant supporting information is included in the manuscript submission,

* There are no issues that prevent the paper from being properly typeset

You will receive further instructions from the production team, including instructions on how to review your proof when it is ready. Please keep in mind that we are working through a large volume of accepted articles, so please give us a few days to review your paper and let you know the next and final steps.

Lastly, if your institution or institutions have a press office, please let them know about your upcoming paper now to help maximize its impact. If they'll be preparing press materials, please inform our press team within the next 48 hours. Your manuscript will remain under strict press embargo until 2 pm Eastern Time on the date of publication. For more information, please contact onepress@plos.org.

You will receive an invoice from PLOS for your publication fee after your manuscript has reached the completed accept phase. If you receive an email requesting payment before acceptance or for any other service, this may be a phishing scheme. Learn how to identify phishing emails and protect your accounts at https://explore.plos.org/phishing.

If we can help with anything else, please email us at customercare@plos.org.

Thank you for submitting your work to PLOS ONE and supporting open access.

Kind regards,

PLOS ONE Editorial Office Staff

on behalf of

Dr. Rajesh Kumar

Academic Editor

PLOS One
